# Supplementary material for: Data Fusion of Fourier Transform Mid-Infrared (MIR) and Near-Infrared (NIR) Spectroscopies to Identify Geographical Origin of Wild Paris polyphylla var. yunnanensis
Source: Molecules. 2019 Jul 13;24(14):2559. doi: 10.3390/molecules24142559 (PMC6680555; doi:10.3390/molecules24142559)
Supplement: Supplementary file 1 [file molecules-24-02559-s001.pdf]

Data Fusion of Fourier Transform Mid-Infrared (MIR) and Near-Infrared (NIR) Spectroscopies to Identify Geographical Origin of Wild *Paris polyphylla* var. *yunnanensis*

Yi-Fei Pei [1,2], Zhi-Tian Zuo [1], Qing-Zhi Zhang [2,*] and Yuan-Zhong Wang [1,*]

[1] Institute of Medicinal Plants, Yunnan Academy of Agricultural Sciences, Kunming 650200, China
[2] College of Traditional Chinese Medicine, Yunnan University of Chinese Medicine, Kunming 650500, China
* Corresponding author: boletus@126.com (Y.-Z.W.); ynkzqz@126.com (Q.-Z.Z.)

Figure captions

Figure S1 Plots of the permutation test of the FT-MIR PLS-DA model.

Figure S2 The parameter optimization of random forest models for mid-level data fusion decision making before variables ranked by permutation accuracy importance: a, n_{tree} of the FT-MIR-RFE dataset; b, m_{try} of the FT-MIR-RFE dataset; c, n_{tree} of the NIR-RFE dataset; d, m_{try} of the NIR-RFE dataset.

Figure S3 The parameter optimization of random forest models for high-level data fusion decision making before variables ranked by permutation accuracy importance: a, n_{tree} of the FT-MIR-PCs dataset; b, m_{try} of the FT-MIR-PCs dataset; c, n_{tree} of the NIR-PCs dataset; d, m_{try} of the NIR-PCs dataset.

Figure S4 The 10-fold cross validation error rates of the RF model for high-level data fusion (sequentially reduce each five variables) based on total *P. yunnanensis* samples: a, FT-MIR dataset; b, NIR dataset.

Figure S5 The parameter optimization of random forest models for high-level data fusion decision making before variables ranked by permutation accuracy importance: a, n_{tree} of the FT-MIR-RFE dataset; b, m_{try} of the FT-MIR-FRE dataset; c, n_{tree} of the NIR-RFE dataset; d, m_{try} of the NIR-RFE dataset.

Figure S6 The parameter optimization of random forest models for high-level data fusion decision making before variables ranked by permutation accuracy importance: a, n_{tree} of the FT-MIR-Bo dataset; b, m_{try} of the FT-MIR-Bo dataset; c, n_{tree} of the NIR-Bo dataset; d, m_{try} of the NIR-Bo dataset.

Figure S7 The important variables of Boruta method and RFE method of random forest models based on the total *P. yunnanensis* samples: a, b, the FT-MIR dataset; c, d, the NIR dataset.

Figure S8 The parameter optimization of random forest models for low-level data fusion decision making after variables ranked by permutation accuracy importance: a, n_{tree} of the FT-MIR-RFE dataset; b, m_{try} of the FT-MIR-RFE dataset; c, n_{tree} of the NIR-RFE dataset; d, m_{try} of the NIR-RFE dataset.

Figure S9 The VIP scores plot of PLS-DA model based on the total *P. yunnanensis* samples.

Figure S10 The parameter optimization of random forest models of mid-level data fusion decision making after variables ranked by permutation accuracy importance: a, n_{tree} of the mid-level-PCs dataset; b, m_{try} of the mid-level-PCs dataset; c, n_{tree} of the mid-level-RFE dataset; d, m_{try} of the mid-level-RFE dataset; e, n_{tree} of the mid-level-Bo dataset; f, m_{try} of the mid-level-Bo dataset.

Table captions

Table S1 The major parameters of raw and preprocessing calibration models based on *P. yunnanensis* samples combined with FT-MIR and NIR spectra.

Table S2 The tree votes of FT-MIR-PCs and NIR-PCs RF model in calibration set.

Table S3 The tree votes of FT-MIR-PCs and NIR-PCs RF model in validation set.

Table S4 The tree votes of FT-MIR-PCs and NIR-PCs PLS-DA model in calibration set.

Table S5 The tree votes of FT-MIR-PCs and NIR-PCs PLS-DA model in validation set.

Table S6 The tree votes of FT-MIR-RFE and NIR-RFE RF model in calibration set.

Table S7 The tree votes of FT-MIR-RFE and NIR-RFE RF model in validation set.

Table S8 The tree votes of FT-MIR-RFE and NIR-RFE PLS-DA model in calibration set.

Table S9 The tree votes of FT-MIR-RFE and NIR-RFE PLS-DA model in validation set.

Table S10 The tree votes of FT-MIR-Bo and NIR-Bo RF model in calibration set.

Table S11 The tree votes of FT-MIR-Bo and NIR-Bo RF model in validation set.

Table S12 The tree votes of FT-MIR-Bo and NIR-Bo PLS-DA model in calibration set.

Table S13 The tree votes of FT-MIR-Bo and NIR-Bo PLS-DA model in validation set.

Table S14 Class assignation of *P. yunnanensis* samples using fuzzy set theory in high-level data fusion.

Table S15 The geographical location of wild *P. yunnanensis* samples.

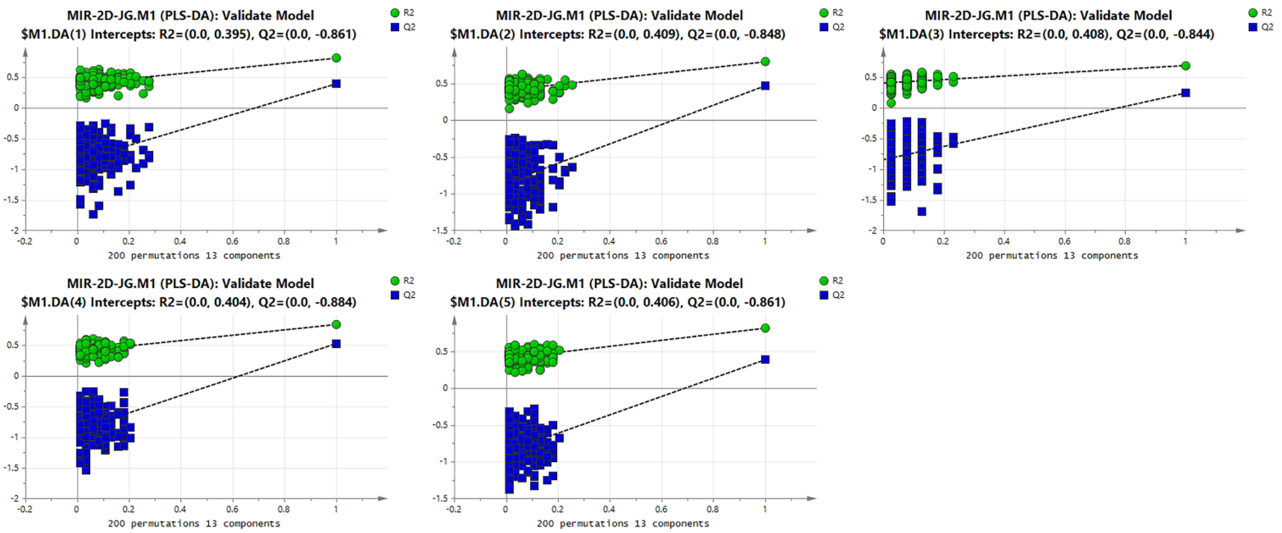
MIR-2D-JG.M1 (PLS-DA): Validate Model
$M1.DA(1) Intercepts: R2=(0.0, 0.395), Q2=(0.0, -0.861)
200 permutations 13 components
MIR-2D-JG.M1 (PLS-DA): Validate Model
$M1.DA(2) Intercepts: R2=(0.0, 0.409), Q2=(0.0, -0.848)
200 permutations 13 components
MIR-2D-JG.M1 (PLS-DA): Validate Model
$M1.DA(3) Intercepts: R2=(0.0, 0.408), Q2=(0.0, -0.844)
200 permutations 13 components
MIR-2D-JG.M1 (PLS-DA): Validate Model
$M1.DA(4) Intercepts: R2=(0.0, 0.404), Q2=(0.0, -0.884)
200 permutations 13 components
MIR-2D-JG.M1 (PLS-DA): Validate Model
$M1.DA(5) Intercepts: R2=(0.0, 0.406), Q2=(0.0, -0.861)
200 permutations 13 components
R2
Q2

Fig. S1

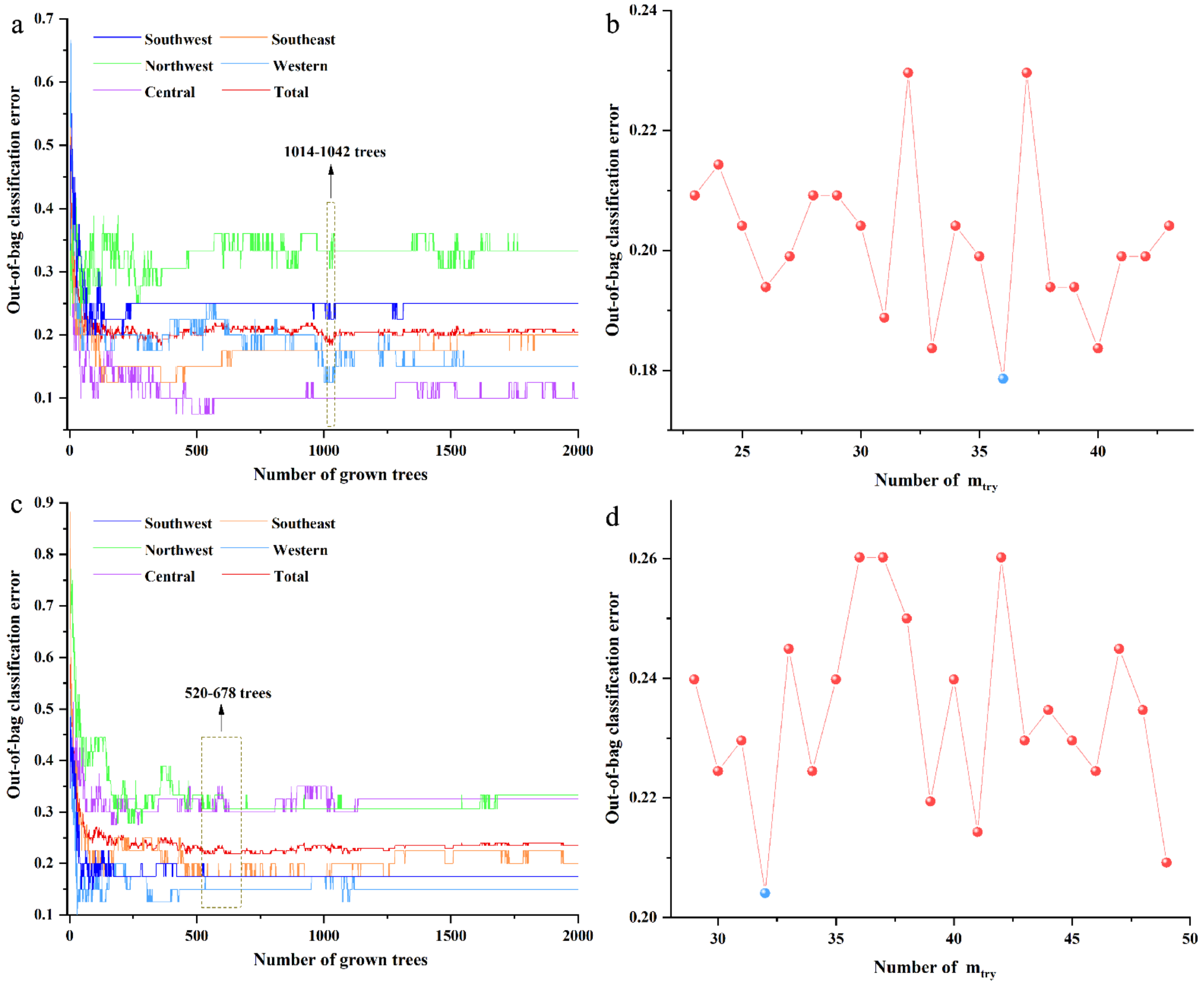
a
Southwest
Southeast
Northwest
Western
Central
Total
1014-1042 trees
Out-of-bag classification error
Number of grown trees
b
Out-of-bag classification error
Number of m_{try}
c
Southwest
Southeast
Northwest
Western
Central
Total
520-678 trees
Out-of-bag classification error
Number of grown trees
d
Out-of-bag classification error
Number of m_{try}

Fig. S2

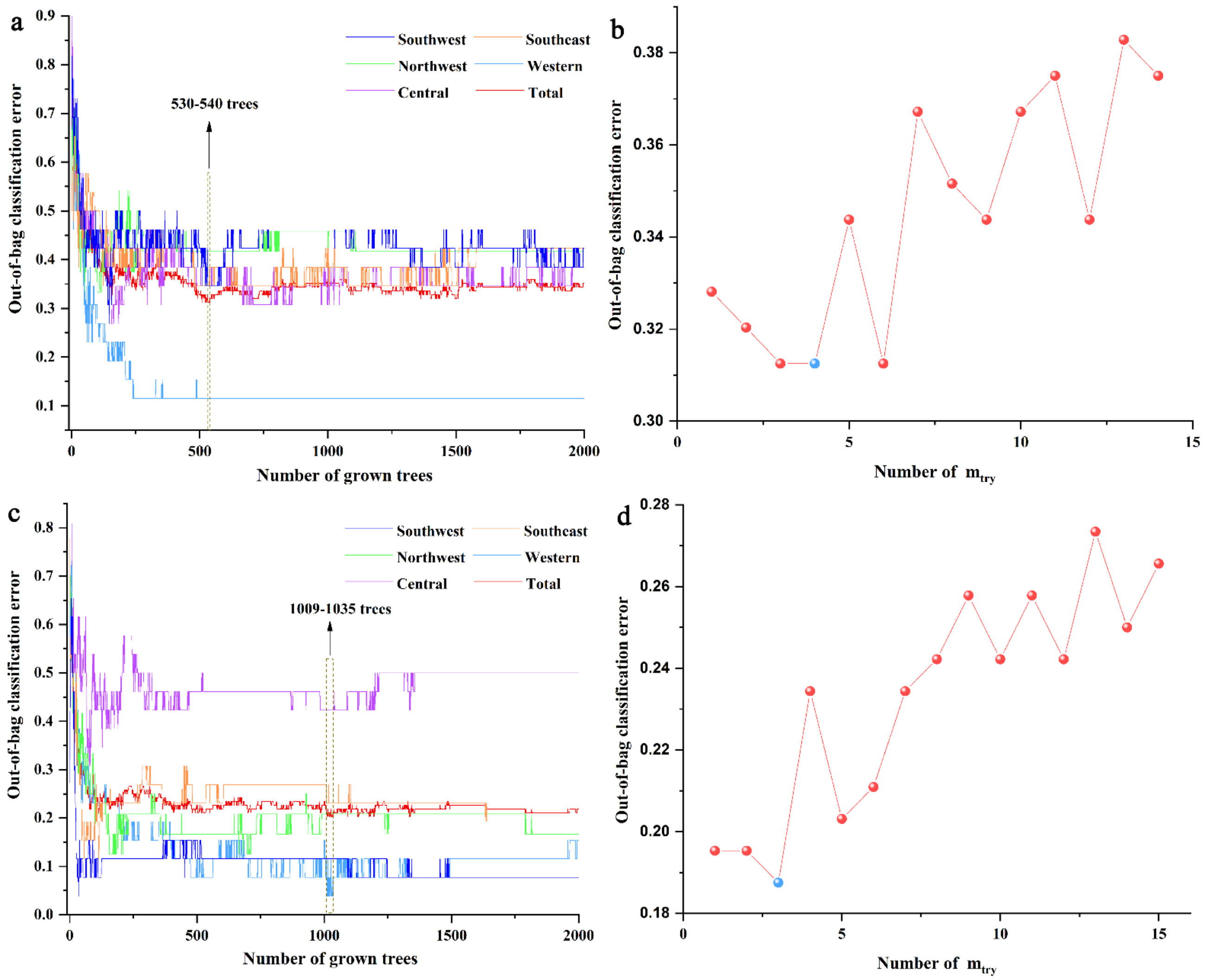
a
b
c
d
Southwest
Southeast
Northwest
Western
Central
Total
530-540 trees
1009-1035 trees
Out-of-bag classification error
Number of grown trees
Number of m_{try}

Fig. S3

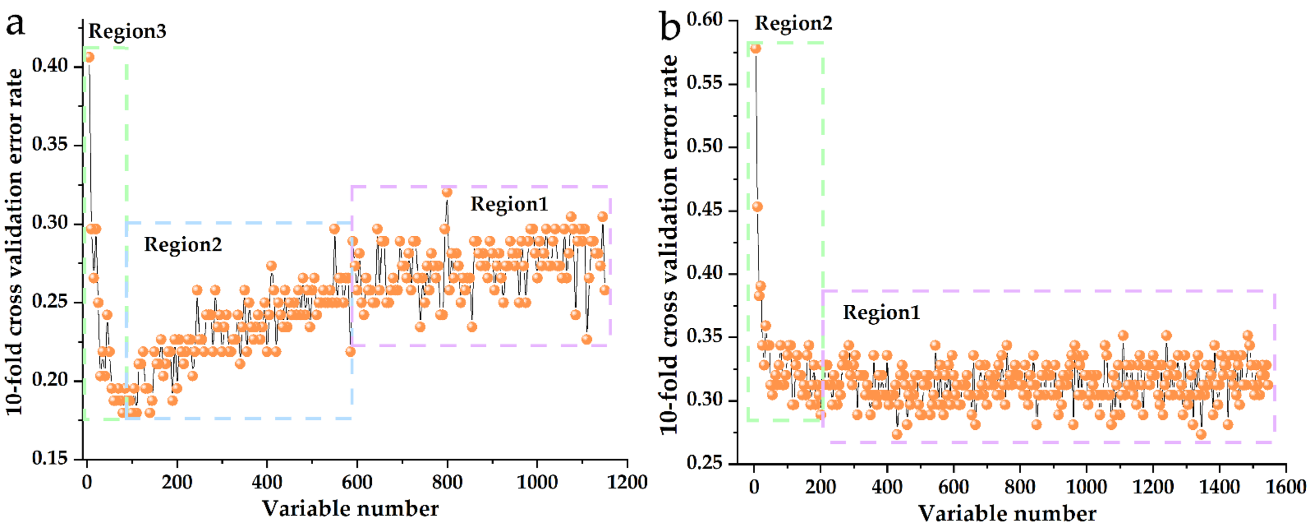

a
Region3
Region2
Region1
10-fold cross validation error rate
0.15
0.20
0.25
0.30
0.35
0.40
0
200
400
600
800
1000
1200
Variable number
b
Region2
Region1
10-fold cross validation error rate
0.25
0.30
0.35
0.40
0.45
0.50
0.55
0.60
0
200
400
600
800
1000
1200
1400
1600
Variable number

Fig. S4

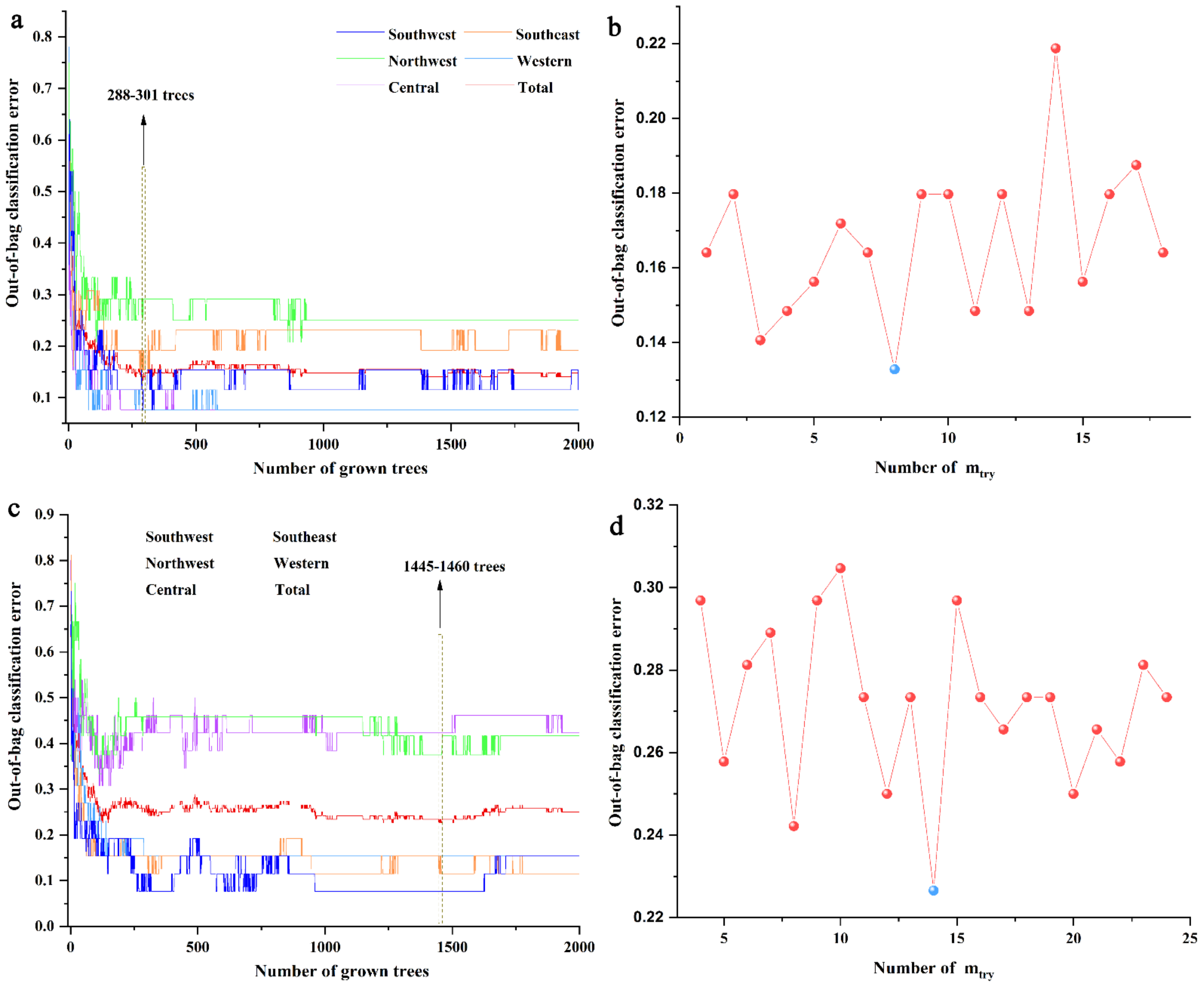
a
Southwest
Southeast
Northwest
Western
Central
Total
288-301 trees
Out-of-bag classification error
Number of grown trees
b
Out-of-bag classification error
Number of m_{try}
c
Southwest
Southeast
Northwest
Western
Central
Total
1445-1460 trees
Out-of-bag classification error
Number of grown trees
d
Out-of-bag classification error
Number of m_{try}

Fig. S5

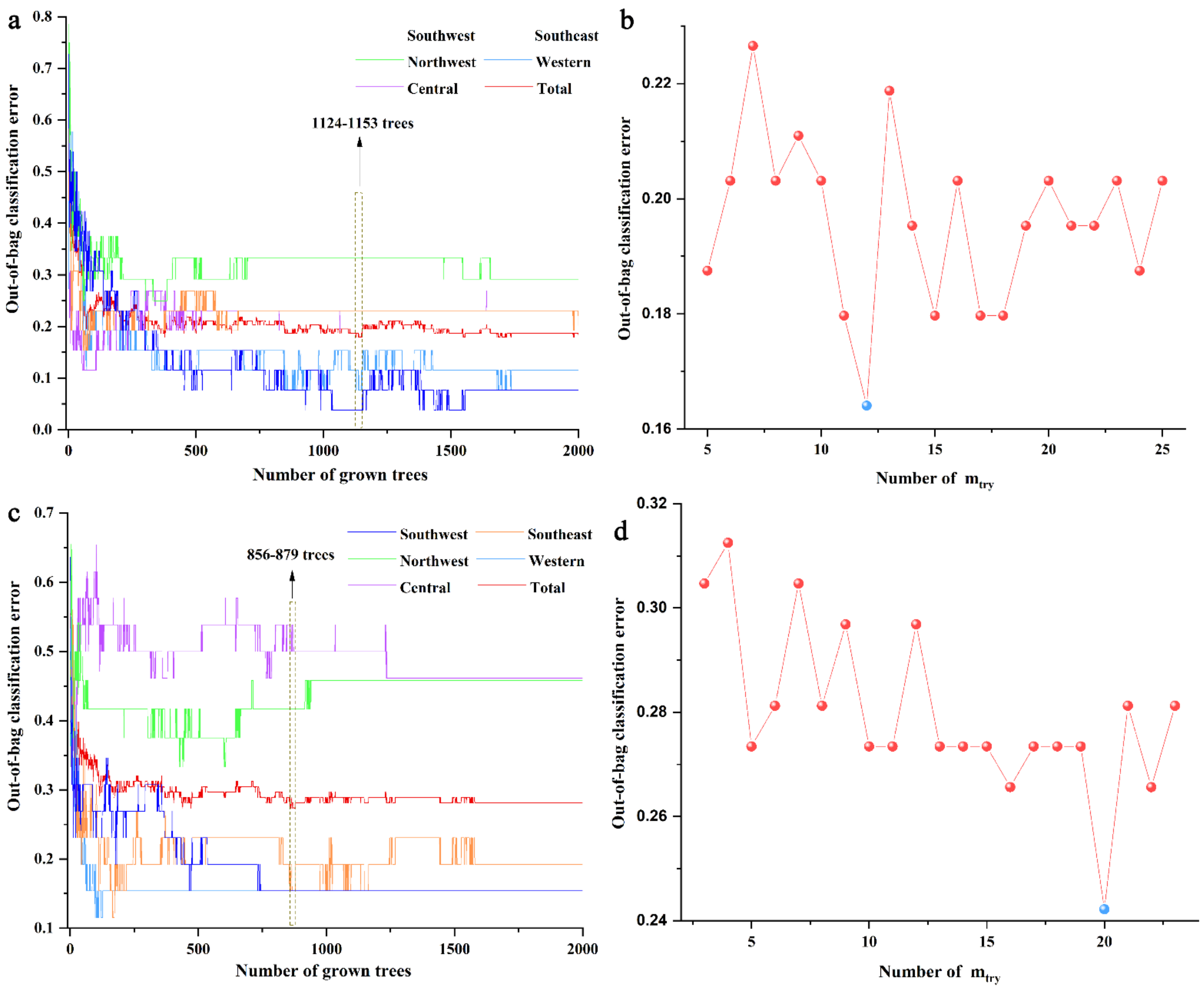
a
Southwest
Southeast
Northwest
Western
Central
Total
1124-1153 trees
Out-of-bag classification error
Number of grown trees
b
Out-of-bag classification error
Number of m_{try}
c
Southwest
Southeast
Northwest
Western
Central
Total
856-879 trees
Out-of-bag classification error
Number of grown trees
d
Out-of-bag classification error
Number of m_{try}

Fig. S6

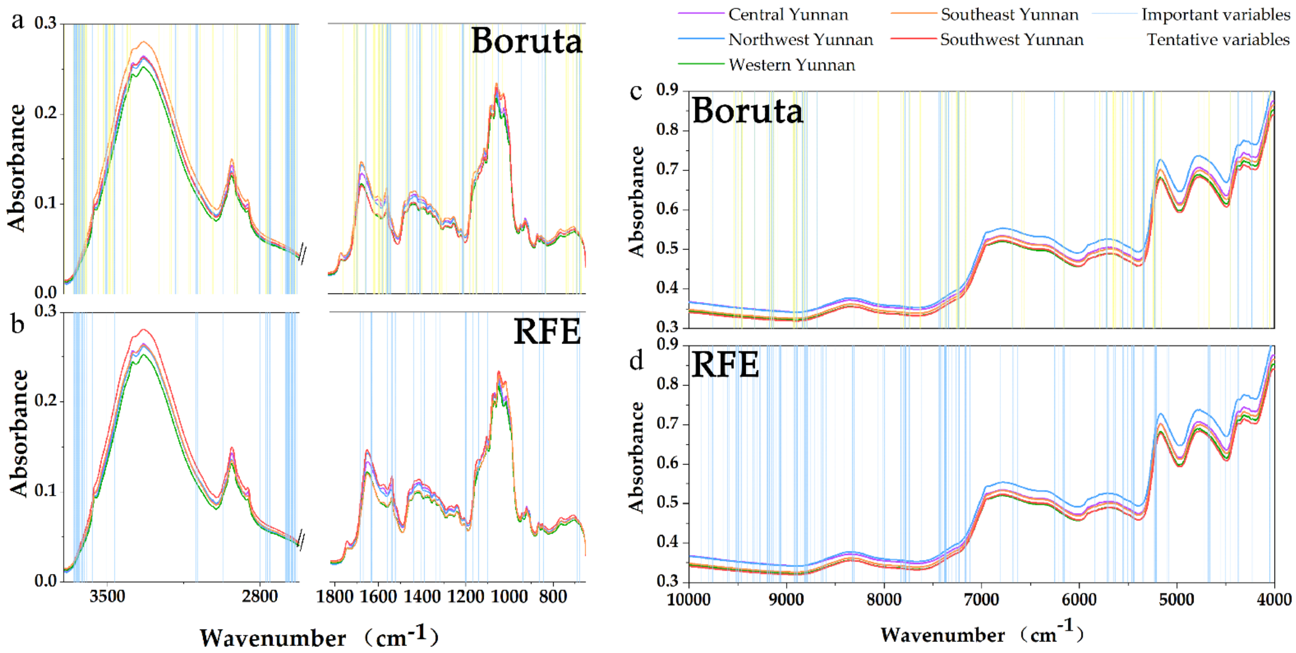

Central Yunnan
Southeast Yunnan
Important variables
Northwest Yunnan
Southwest Yunnan
Tentative variables
Western Yunnan
a
b
c
d
Boruta
RFE
Boruta
RFE
Absorbance
Wavenumber （cm-1）

Fig. S7

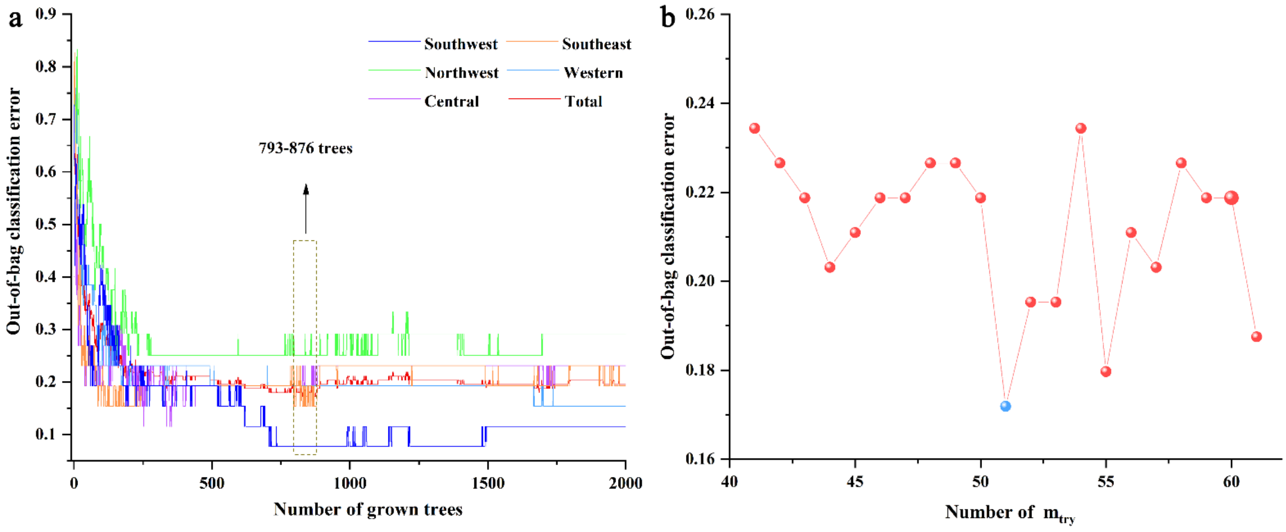

a
Out-of-bag classification error
0.9
0.8
0.7
0.6
0.5
0.4
0.3
0.2
0.1
Southwest
Southeast
Northwest
Western
Central
Total
793-876 trees
0
500
1000
1500
2000
Number of grown trees
b
0.26
0.24
0.22
0.20
0.18
0.16
40
45
50
55
60
Number of m_{try}

Fig. S8

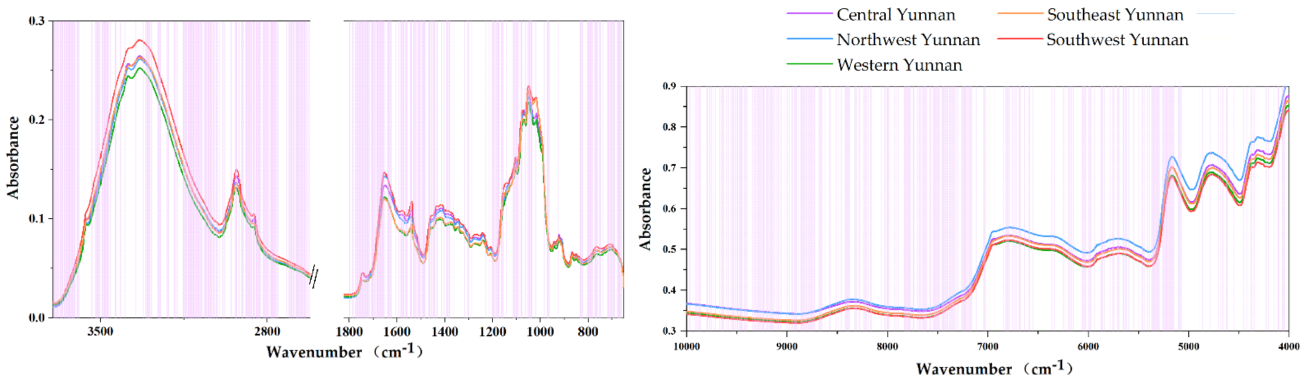
Central Yunnan
Southeast Yunnan
Northwest Yunnan
Southwest Yunnan
Western Yunnan
Absorbance
0.3
0.2
0.1
0.0
3500
2800
1800
1600
1400
1200
1000
800
Wavenumber （cm-1）
0.9
0.8
0.7
0.6
0.5
0.4
0.3
10000
9000
8000
7000
6000
5000
4000
Absorbance
Wavenumber （cm-1）

Fig. S9

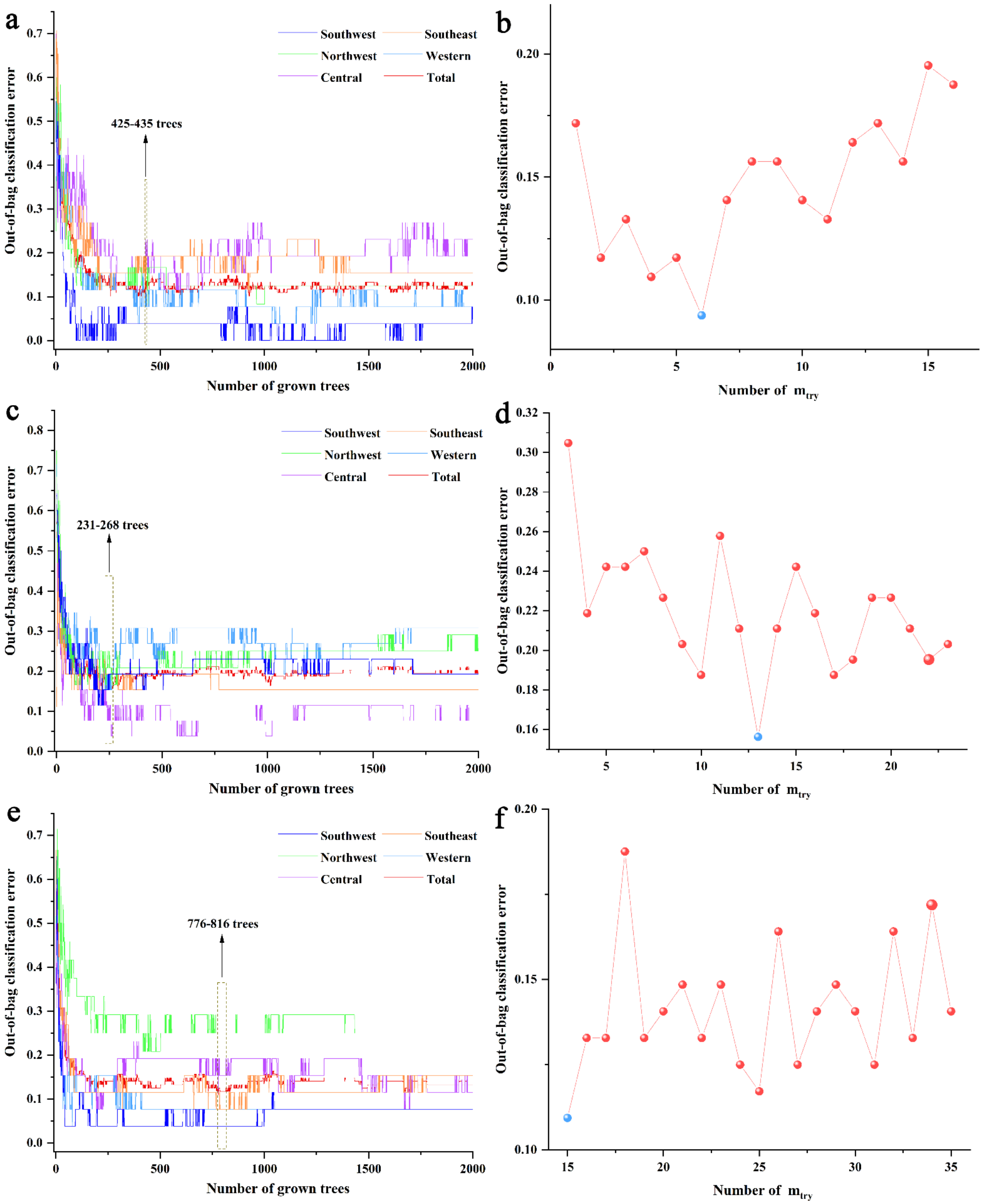
a
b
c
d
e
f
Southwest
Southeast
Northwest
Western
Central
Total
425-435 trees
231-268 trees
776-816 trees
Out-of-bag classification error
Number of grown trees
Number of m_{try}

Fig. S10

Table S1

Data sources	Preprocessing	R^2	Q^2	RMSEE	RMSECV	Accuracy
FT-MIR	Raw	0.051	0.0097	0.379794	0.390348	34.38%
	SNV	0.079	0.0173	0.388073	0.396241	39.06%
	FD	0.072	0.0379	0.387724	0.391868	30.47%
	**SD**	**0.747**	**0.385**	**0.211095**	**0.332652**	**97.66%**
	SNV-FD	0.48	0.1691	0.301191	0.371033	78.13%
	SNV-SD	0.522	0.28	0.284848	0.348765	85.29%
NIR	Raw	0.037	0.0229	0.395604	0.395266	28.13%
	SNV	0.068	0.0364	0.390668	0.392528	26.56%
	FD	0.047	0.0005	0.393084	0.399797	32.03%
	**SD**	**0.879**	**0.628**	**0.145553**	**0.257344**	**100%**
	SNV-FD	0.333	0.143	0.335555	0.369848	67.19%
	SNV-SD	0.781	0.457	0.194624	0.300091	98.44%

Table S2

Sample number	Class label	FT-MIR-PCs tree votes (Calibration set)					NIR-PCs tree votes (Calibration set)				
		Class 1	Class 2	Class 3	Class 4	Class 5	Class 1	Class 2	Class 3	Class 4	Class 5
36	1	0.302703	0.091892	0.259459	0.145946	0.2	0.387597	0.142119	0.111111	0.111111	0.248062
33	1	0.380711	0.248731	0.177665	0.13198	0.060914	0.444149	0.180851	0.130319	0.090426	0.154255
12	1	0.317204	0.107527	0.064516	0.376344	0.134409	0.280519	0.155844	0.246753	0.2	0.116883
1	1	0.453608	0.231959	0.154639	0.046392	0.113402	0.37931	0.212202	0.106101	0.206897	0.095491
2	1	0.46114	0.098446	0.139896	0.124352	0.176166	0.180203	0.126904	0.076142	0.121827	0.494924
20	1	0.309392	0.143646	0.121547	0.292818	0.132597	0.205962	0.262873	0.279133	0.159892	0.092141
19	1	0.35122	0.117073	0.131707	0.165854	0.234146	0.325137	0.103825	0.142077	0.125683	0.303279
27	1	0.304124	0.175258	0.087629	0.21134	0.221649	0.360335	0.117318	0.086592	0.164804	0.27095
16	1	0.257732	0.103093	0.164948	0.159794	0.314433	0.289125	0.28382	0.177719	0.148541	0.100796
31	1	0.136126	0.104712	0.209424	0.162304	0.387435	0.227273	0.18984	0.352941	0.122995	0.106952
3	1	0.386139	0.227723	0.123762	0.113861	0.148515	0.327078	0.112601	0.088472	0.160858	0.310992
18	1	0.289216	0.034314	0.117647	0.323529	0.235294	0.208226	0.154242	0.197943	0.303342	0.136247
9	1	0.412698	0.042328	0.15873	0.31746	0.068783	0.37883	0.133705	0.181058	0.089136	0.21727
26	1	0.192513	0.085561	0.245989	0.304813	0.171123	0.420485	0.142857	0.113208	0.091644	0.231806
25	1	0.317073	0.219512	0.136585	0.209756	0.117073	0.246269	0.114428	0.156716	0.134328	0.348259
23	1	0.282051	0.153846	0.148718	0.307692	0.107692	0.323529	0.104278	0.101604	0.117647	0.352941
38	1	0.169312	0.148148	0.407407	0.089947	0.185185	0.338843	0.118457	0.181818	0.22865	0.132231
14	1	0.311224	0.153061	0.280612	0.112245	0.142857	0.25641	0.356125	0.236467	0.099715	0.051282
40	1	0.238579	0.086294	0.111675	0.116751	0.446701	0.287599	0.324538	0.137203	0.16095	0.08971
5	1	0.417112	0.13369	0.15508	0.203209	0.090909	0.390123	0.069136	0.096296	0.165432	0.279012
29	1	0.22335	0.233503	0.192893	0.121827	0.228426	0.306407	0.122563	0.122563	0.122563	0.325905
39	1	0.413043	0.092391	0.358696	0.065217	0.070652	0.456395	0.110465	0.075581	0.194767	0.162791
17	1	0.378378	0.102703	0.145946	0.156757	0.216216	0.193211	0.130548	0.185379	0.362924	0.127937
7	1	0.487562	0.134328	0.064677	0.149254	0.164179	0.433915	0.154613	0.114713	0.15212	0.144638
21	1	0.472906	0.137931	0.083744	0.182266	0.123153	0.176152	0.298103	0.289973	0.170732	0.065041
11	1	0.2723	0.126761	0.267606	0.126761	0.206573	0.344221	0.123116	0.153266	0.18593	0.193467
62	2	0.092233	0.543689	0.242718	0.106796	0.014563	0.198473	0.335878	0.167939	0.165394	0.132316
59	2	0.160194	0.446602	0.106796	0.218447	0.067961	0.186813	0.299451	0.17033	0.252747	0.090659
56	2	0.045685	0.426396	0.208122	0.177665	0.142132	0.177285	0.33795	0.144044	0.254848	0.085873
72	2	0.07767	0.509709	0.063107	0.184466	0.165049	0.293963	0.351706	0.11811	0.186352	0.049869
44	2	0.084577	0.512438	0.104478	0.079602	0.218905	0.148615	0.428212	0.146096	0.123426	0.153652
45	2	0.063415	0.541463	0.068293	0.146341	0.180488	0.174731	0.446237	0.158602	0.110215	0.110215
63	2	0.378238	0.15544	0.202073	0.19171	0.072539	0.150127	0.279898	0.201018	0.21374	0.155216
41	2	0.243094	0.165746	0.149171	0.276243	0.165746	0.09949	0.387755	0.145408	0.260204	0.107143
51	2	0.162921	0.275281	0.252809	0.162921	0.146067	0.143603	0.438642	0.13577	0.151436	0.130548
79	2	0.185567	0.469072	0.113402	0.128866	0.103093	0.149584	0.296399	0.213296	0.238227	0.102493
48	2	0.122066	0.43662	0.201878	0.131455	0.107981	0.160428	0.409091	0.136364	0.203209	0.090909
76	2	0.230366	0.308901	0.225131	0.167539	0.068063	0.123324	0.348525	0.230563	0.179625	0.117962
68	2	0.071795	0.738462	0.041026	0.051282	0.097436	0.099738	0.430446	0.110236	0.267717	0.091864
55	2	0.06278	0.591928	0.147982	0.134529	0.06278	0.174033	0.364641	0.162983	0.135359	0.162983
61	2	0.122905	0.145251	0.167598	0.312849	0.251397	0.155172	0.428161	0.123563	0.175287	0.117816
74	2	0.072917	0.619792	0.083333	0.083333	0.140625	0.13577	0.475196	0.093995	0.177546	0.117493
70	2	0.152542	0.316384	0.124294	0.242938	0.163842	0.386301	0.317808	0.090411	0.167123	0.038356
54	2	0.048913	0.581522	0.065217	0.168478	0.13587	0.389205	0.210227	0.15625	0.153409	0.090909
66	2	0.115183	0.329843	0.099476	0.198953	0.256545	0.14578	0.350384	0.15601	0.225064	0.122762
64	2	0.181347	0.264249	0.170984	0.227979	0.15544	0.131783	0.315245	0.198966	0.22739	0.126615

57	2	0.260204	0.352041	0.117347	0.091837	0.178571	0.109453	0.400498	0.273632	0.077114	0.139303
75	2	0.113402	0.386598	0.072165	0.092784	0.335052	0.227778	0.427778	0.088889	0.169444	0.086111
47	2	0.215311	0.334928	0.253589	0.057416	0.138756	0.219144	0.43073	0.115869	0.173804	0.060453
77	2	0.073298	0.413613	0.230366	0.13089	0.151832	0.119792	0.463542	0.135417	0.111979	0.169271
53	2	0.258537	0.35122	0.102439	0.107317	0.180488	0.103093	0.556701	0.085052	0.154639	0.100515
50	2	0.041026	0.45641	0.235897	0.14359	0.123077	0.208451	0.256338	0.242254	0.149296	0.143662
101	3	0.167513	0.172589	0.248731	0.208122	0.203046	0.164179	0.256219	0.208955	0.251244	0.119403
91	3	0.103093	0.159794	0.618557	0.07732	0.041237	0.123656	0.091398	0.537634	0.145161	0.102151
90	3	0.213592	0.184466	0.165049	0.271845	0.165049	0.179949	0.159383	0.365039	0.195373	0.100257
87	3	0.309524	0.195238	0.166667	0.180952	0.147619	0.098191	0.20155	0.281654	0.235142	0.183463
99	3	0.107317	0.102439	0.253659	0.141463	0.395122	0.252604	0.377604	0.135417	0.158854	0.075521
114	3	0.216931	0.354497	0.121693	0.185185	0.121693	0.085052	0.180412	0.407216	0.257732	0.069588
116	3	0.340782	0.139665	0.24581	0.145251	0.128492	0.183099	0.149296	0.140845	0.309859	0.216901
102	3	0.149733	0.058824	0.315508	0.240642	0.235294	0.19571	0.179625	0.313673	0.152815	0.158177
104	3	0.183099	0.164319	0.43662	0.131455	0.084507	0.177378	0.102828	0.583548	0.051414	0.084833
83	3	0.149321	0.235294	0.334842	0.095023	0.18552	0.220513	0.269231	0.274359	0.107692	0.128205
110	3	0.145078	0.11399	0.393782	0.145078	0.202073	0.191176	0.188725	0.397059	0.107843	0.115196
108	3	0.368687	0.065657	0.30303	0.060606	0.20202	0.182292	0.119792	0.476563	0.117188	0.104167
81	3	0.227053	0.072464	0.396135	0.207729	0.096618	0.282667	0.122667	0.261333	0.232	0.101333
107	3	0.073298	0.172775	0.078534	0.141361	0.534031	0.164804	0.150838	0.458101	0.164804	0.061453
112	3	0.280788	0.093596	0.384236	0.108374	0.133005	0.210667	0.149333	0.341333	0.181333	0.117333
113	3	0.151832	0.057592	0.664921	0.052356	0.073298	0.222222	0.143631	0.233062	0.181572	0.219512
97	3	0.204545	0.125	0.409091	0.159091	0.102273	0.305851	0.167553	0.316489	0.119681	0.090426
85	3	0.102041	0.071429	0.27551	0.346939	0.204082	0.19685	0.183727	0.496063	0.081365	0.041995
109	3	0.079787	0.244681	0.37234	0.175532	0.12766	0.193211	0.276762	0.318538	0.091384	0.120104
105	3	0.033493	0.105263	0.287081	0.162679	0.411483	0.104439	0.29765	0.375979	0.122715	0.099217
100	3	0.315789	0.105263	0.357895	0.126316	0.094737	0.172237	0.136247	0.375321	0.251928	0.064267
111	3	0.177419	0.069892	0.639785	0.069892	0.043011	0.253298	0.081794	0.242744	0.07124	0.350923
96	3	0.067708	0.072917	0.270833	0.380208	0.208333	0.114441	0.122616	0.378747	0.245232	0.138965
106	3	0.17	0.125	0.535	0.115	0.055	0.102828	0.105398	0.48329	0.125964	0.182519
152	4	0.219626	0.088785	0.392523	0.21028	0.088785	0.197333	0.357333	0.208	0.157333	0.08
121	4	0.245989	0.240642	0.085561	0.294118	0.13369	0.188366	0.166205	0.144044	0.432133	0.069252
125	4	0.116751	0.13198	0.126904	0.395939	0.228426	0.132468	0.21039	0.150649	0.428571	0.077922
128	4	0.134503	0.181287	0.076023	0.374269	0.233918	0.242021	0.12766	0.138298	0.223404	0.268617
145	4	0.091429	0.142857	0.068571	0.674286	0.022857	0.143959	0.195373	0.164524	0.401028	0.095116
156	4	0.233918	0.064327	0.315789	0.327485	0.05848	0.170732	0.271003	0.127371	0.276423	0.154472
126	4	0.149485	0.139175	0.149485	0.510309	0.051546	0.102493	0.155125	0.171745	0.373961	0.196676
131	4	0.156627	0.216867	0.066265	0.5	0.060241	0.316547	0.11271	0.134293	0.297362	0.139089
117	4	0.229592	0.214286	0.112245	0.239796	0.204082	0.126649	0.065963	0.184697	0.522427	0.100264
122	4	0.220994	0.082873	0.143646	0.20442	0.348066	0.29199	0.093023	0.077519	0.315245	0.222222
149	4	0.216749	0.147783	0.226601	0.275862	0.133005	0.176923	0.151282	0.169231	0.402564	0.1
153	4	0.111111	0.144928	0.072464	0.299517	0.371981	0.140212	0.097884	0.10582	0.526455	0.12963
146	4	0.306011	0.092896	0.136612	0.360656	0.103825	0.172414	0.114058	0.167109	0.427056	0.119363
123	4	0.084906	0.103774	0.042453	0.726415	0.042453	0.138272	0.234568	0.160494	0.367901	0.098765
139	4	0.098039	0.127451	0.029412	0.686275	0.058824	0.2675	0.1775	0.0875	0.2825	0.185
140	4	0.165	0.075	0.155	0.465	0.14	0.150754	0.125628	0.135678	0.505025	0.082915
137	4	0.110553	0.175879	0.341709	0.160804	0.211055	0.193029	0.227882	0.209115	0.19571	0.174263
144	4	0.218579	0.125683	0.131148	0.251366	0.273224	0.18799	0.214099	0.18799	0.331593	0.078329
130	4	0.043689	0.208738	0.053398	0.679612	0.014563	0.245946	0.178378	0.113514	0.272973	0.189189

118	4	0.068421	0.363158	0.184211	0.126316	0.257895	0.210227	0.102273	0.079545	0.420455	0.1875
148	4	0.346535	0.10396	0.123762	0.29703	0.128713	0.212598	0.16273	0.186352	0.283465	0.154856
134	4	0.053922	0.137255	0.191176	0.573529	0.044118	0.108861	0.182278	0.174684	0.425316	0.108861
141	4	0.095745	0.180851	0.101064	0.340426	0.281915	0.079897	0.10567	0.141753	0.556701	0.115979
136	4	0.126168	0.140187	0.121495	0.551402	0.060748	0.08094	0.127937	0.234987	0.472585	0.083551
154	4	0.076142	0.035533	0.401015	0.208122	0.279188	0.259804	0.176471	0.166667	0.32598	0.071078
129	4	0.321053	0.094737	0.152632	0.242105	0.189474	0.245399	0.300613	0.113497	0.260736	0.079755
192	5	0.242424	0.161616	0.141414	0.151515	0.30303	0.194373	0.038363	0.086957	0.076726	0.603581
171	5	0.192708	0.192708	0.104167	0.25	0.260417	0.171662	0.174387	0.103542	0.13624	0.414169
169	5	0.253886	0.253886	0.129534	0.134715	0.227979	0.124309	0.154696	0.19337	0.323204	0.20442
162	5	0.284153	0.163934	0.15847	0.103825	0.289617	0.192	0.165333	0.189333	0.074667	0.378667
182	5	0.231959	0.195876	0.072165	0.113402	0.386598	0.262087	0.07888	0.076336	0.117048	0.465649
190	5	0.15625	0.130208	0.140625	0.328125	0.244792	0.229008	0.139949	0.155216	0.068702	0.407125
167	5	0.234637	0.078212	0.413408	0.106145	0.167598	0.32337	0.141304	0.127717	0.084239	0.32337
178	5	0.254808	0.033654	0.350962	0.100962	0.259615	0.213158	0.123684	0.073684	0.155263	0.434211
196	5	0.161458	0.166667	0.088542	0.15625	0.427083	0.251337	0.157754	0.109626	0.093583	0.387701
165	5	0.162437	0.055838	0.258883	0.253807	0.269036	0.283019	0.072776	0.156334	0.102426	0.385445
187	5	0.171429	0.22381	0.304762	0.07619	0.22381	0.137566	0.164021	0.121693	0.156085	0.420635
191	5	0.071429	0.132653	0.367347	0.188776	0.239796	0.161369	0.158924	0.070905	0.122249	0.486553
184	5	0.143646	0.116022	0.226519	0.276243	0.237569	0.160428	0.136364	0.149733	0.09893	0.454545
161	5	0.070707	0.30303	0.207071	0.222222	0.19697	0.12931	0.043103	0.123563	0.097701	0.606322
180	5	0.266667	0.097436	0.092308	0.097436	0.446154	0.2375	0.1875	0.1475	0.115	0.3125
175	5	0.072917	0.244792	0.1875	0.239583	0.255208	0.265823	0.108861	0.116456	0.063291	0.44557
179	5	0.258216	0.140845	0.187793	0.13615	0.276995	0.179551	0.104738	0.087282	0.229426	0.399002
168	5	0.252475	0.118812	0.044554	0.118812	0.465347	0.135359	0.093923	0.104972	0.129834	0.535912
189	5	0.170854	0.155779	0.160804	0.090452	0.422111	0.241935	0.083333	0.051075	0.27957	0.344086
173	5	0.178571	0.163265	0.122449	0.183673	0.352041	0.181333	0.058667	0.117333	0.12	0.522667
194	5	0.156566	0.106061	0.287879	0.252525	0.19697	0.198413	0.089947	0.108466	0.190476	0.412698
195	5	0.133333	0.138889	0.1	0.111111	0.516667	0.168449	0.131016	0.069519	0.176471	0.454545
170	5	0.09	0.07	0.035	0.135	0.67	0.154255	0.087766	0.125	0.146277	0.486702
174	5	0.112745	0.112745	0.107843	0.127451	0.539216	0.12782	0.072682	0.110276	0.157895	0.531328
163	5	0.190955	0.125628	0.090452	0.251256	0.341709	0.267857	0.066327	0.15051	0.089286	0.42602
181	5	0.161137	0.090047	0.21327	0.123223	0.412322	0.076923	0.07967	0.085165	0.068681	0.68956

Table S3

Sample number	Class label	FT-MIR-PCs tree votes (Validation set)					NIR-PCs tree votes (Validation set)				
		Class 1	Class 2	Class 3	Class 4	Class 5	Class 1	Class 2	Class 3	Class 4	Class 5
4	1	0.500935	0.104673	0.069159	0.263551	0.061682	0.33301	0.072816	0.140777	0.108738	0.34466
6	1	0.246729	0.149533	0.084112	0.321495	0.198131	0.360194	0.183495	0.132039	0.280583	0.043689
8	1	0.568224	0.063551	0.061682	0.171963	0.134579	0.368932	0.112621	0.147573	0.231068	0.139806
10	1	0.379439	0.11028	0.170093	0.233645	0.106542	0.380583	0.058252	0.174757	0.145631	0.240777
13	1	0.435514	0.069159	0.093458	0.26729	0.134579	0.405825	0.085437	0.128155	0.131068	0.249515
15	1	0.345794	0.115888	0.114019	0.269159	0.15514	0.415534	0.164078	0.23301	0.135922	0.051456
22	1	0.28785	0.11028	0.13271	0.254206	0.214953	0.570874	0.096117	0.11068	0.070874	0.151456
24	1	0.271028	0.057944	0.239252	0.256075	0.175701	0.541748	0.136893	0.092233	0.078641	0.150485
28	1	0.327103	0.080374	0.171963	0.149533	0.271028	0.531068	0.078641	0.136893	0.07767	0.175728
30	1	0.717757	0.059813	0.093458	0.072897	0.056075	0.526214	0.07767	0.14466	0.085437	0.166019
32	1	0.616822	0.11215	0.143925	0.063551	0.063551	0.530097	0.084466	0.096117	0.080583	0.208738
34	1	0.375701	0.082243	0.149533	0.08785	0.304673	0.562136	0.070874	0.15534	0.084466	0.127184
35	1	0.327103	0.065421	0.31028	0.095327	0.201869	0.473786	0.162136	0.04466	0.100971	0.218447
37	1	0.413084	0.099065	0.192523	0.123364	0.171963	0.561165	0.070874	0.065049	0.136893	0.166019
42	2	0.11215	0.383178	0.168224	0.140187	0.196262	0.364078	0.274757	0.109709	0.176699	0.074757
43	2	0.071028	0.566355	0.082243	0.160748	0.119626	0.192233	0.307767	0.105825	0.292233	0.101942
46	2	0.095327	0.471028	0.063551	0.157009	0.213084	0.149515	0.427184	0.081553	0.22233	0.119417
49	2	0.078505	0.633645	0.158879	0.039252	0.08972	0.166019	0.563107	0.090291	0.126214	0.054369
52	2	0.074766	0.652336	0.093458	0.046729	0.13271	0.151456	0.399029	0.114563	0.262136	0.072816
58	2	0.085981	0.661682	0.085981	0.052336	0.114019	0.218447	0.34466	0.170874	0.138835	0.127184
60	2	0.237383	0.347664	0.072897	0.166355	0.175701	0.214563	0.480583	0.078641	0.147573	0.078641
65	2	0.168224	0.500935	0.100935	0.08972	0.140187	0.152427	0.480583	0.076699	0.156311	0.133981
67	2	0.061682	0.669159	0.076636	0.085981	0.106542	0.137864	0.449515	0.115534	0.239806	0.057282
69	2	0.162617	0.585047	0.104673	0.080374	0.06729	0.134951	0.538835	0.150485	0.100971	0.074757
71	2	0.143925	0.405607	0.299065	0.076636	0.074766	0.133981	0.535922	0.07767	0.170874	0.081553
73	2	0.100935	0.504673	0.100935	0.091589	0.201869	0.186408	0.431068	0.190291	0.109709	0.082524
78	2	0.11028	0.575701	0.056075	0.106542	0.151402	0.131068	0.523301	0.158252	0.114563	0.072816
80	2	0.198131	0.297196	0.061682	0.203738	0.239252	0.164078	0.349515	0.160194	0.108738	0.217476
82	3	0.082243	0.048598	0.809346	0.024299	0.035514	0.260194	0.174757	0.308738	0.146602	0.109709
84	3	0.084112	0.059813	0.672897	0.11215	0.071028	0.076699	0.129126	0.56699	0.158252	0.068932
86	3	0.100935	0.041121	0.723364	0.084112	0.050467	0.112621	0.153398	0.42233	0.21068	0.100971
88	3	0.039252	0.069159	0.55514	0.076636	0.259813	0.141748	0.136893	0.51068	0.097087	0.113592
89	3	0.04486	0.158879	0.368224	0.181308	0.246729	0.101942	0.087379	0.582524	0.118447	0.109709
92	3	0.076636	0.095327	0.706542	0.078505	0.042991	0.204854	0.146602	0.365049	0.167961	0.115534
93	3	0.108411	0.084112	0.568224	0.166355	0.072897	0.113592	0.102913	0.530097	0.115534	0.137864
94	3	0.097196	0.059813	0.64486	0.121495	0.076636	0.203883	0.17767	0.254369	0.130097	0.233981
95	3	0.143925	0.143925	0.581308	0.082243	0.048598	0.11068	0.132039	0.528155	0.092233	0.136893
98	3	0.170093	0.190654	0.269159	0.224299	0.145794	0.195146	0.114563	0.465049	0.084466	0.140777
103	3	0.085981	0.04486	0.536449	0.196262	0.136449	0.159223	0.092233	0.423301	0.2	0.125243
115	3	0.097196	0.181308	0.414953	0.183178	0.123364	0.097087	0.147573	0.472816	0.18835	0.094175
119	4	0.074766	0.106542	0.082243	0.71028	0.026168	0.121359	0.13301	0.133981	0.42233	0.18932
120	4	0.056075	0.170093	0.063551	0.657944	0.052336	0.158252	0.092233	0.131068	0.478641	0.139806
124	4	0.114019	0.170093	0.082243	0.588785	0.04486	0.207767	0.082524	0.151456	0.492233	0.066019
127	4	0.033645	0.128972	0.031776	0.757009	0.048598	0.134951	0.159223	0.232039	0.378641	0.095146
132	4	0.04486	0.162617	0.048598	0.663551	0.080374	0.267961	0.148544	0.118447	0.374757	0.090291
133	4	0.085981	0.127103	0.042991	0.680374	0.063551	0.100971	0.196117	0.094175	0.558252	0.050485

135	4	0.06729	0.213084	0.085981	0.257944	0.375701	0.151456	0.126214	0.112621	0.51068	0.099029
138	4	0.13271	0.115888	0.104673	0.508411	0.138318	0.179612	0.21068	0.179612	0.373786	0.056311
142	4	0.076636	0.071028	0.186916	0.53271	0.13271	0.108738	0.284466	0.106796	0.41165	0.08835
143	4	0.059813	0.026168	0.28785	0.549533	0.076636	0.198058	0.18835	0.190291	0.31165	0.11165
147	4	0.186916	0.093458	0.054206	0.482243	0.183178	0.161165	0.139806	0.12233	0.423301	0.153398
150	4	0.209346	0.06729	0.128972	0.394393	0.2	0.190291	0.12233	0.107767	0.497087	0.082524
151	4	0.142056	0.108411	0.091589	0.558879	0.099065	0.193204	0.139806	0.095146	0.421359	0.150485
155	4	0.078505	0.054206	0.549533	0.241121	0.076636	0.190291	0.220388	0.074757	0.363107	0.151456
157	5	0.074766	0.056075	0.125234	0.063551	0.680374	0.181553	0.141748	0.13301	0.128155	0.415534
158	5	0.106542	0.056075	0.140187	0.136449	0.560748	0.152427	0.079612	0.150485	0.251456	0.366019
159	5	0.185047	0.074766	0.076636	0.140187	0.523364	0.17767	0.093204	0.116505	0.162136	0.450485
160	5	0.063551	0.031776	0.170093	0.117757	0.616822	0.179612	0.103883	0.08835	0.127184	0.500971
164	5	0.072897	0.063551	0.074766	0.185047	0.603738	0.142718	0.070874	0.138835	0.171845	0.475728
166	5	0.078505	0.11215	0.160748	0.08785	0.560748	0.194175	0.083495	0.067961	0.186408	0.467961
172	5	0.171963	0.095327	0.145794	0.181308	0.405607	0.2	0.058252	0.091262	0.072816	0.57767
176	5	0.157009	0.104673	0.173832	0.226168	0.338318	0.17767	0.127184	0.08835	0.151456	0.45534
177	5	0.235514	0.13271	0.127103	0.078505	0.426168	0.082524	0.063107	0.109709	0.062136	0.682524
183	5	0.123364	0.084112	0.057944	0.080374	0.654206	0.124272	0.091262	0.092233	0.043689	0.648544
185	5	0.084112	0.039252	0.084112	0.024299	0.768224	0.182524	0.070874	0.072816	0.058252	0.615534
186	5	0.123364	0.095327	0.042991	0.136449	0.601869	0.143689	0.108738	0.062136	0.046602	0.638835
188	5	0.143925	0.106542	0.164486	0.085981	0.499065	0.218447	0.057282	0.123301	0.078641	0.52233
193	5	0.078505	0.130841	0.080374	0.102804	0.607477	0.116505	0.095146	0.064078	0.061165	0.663107

Table S4

Sample number	Class label	FT-MIR-PCs tree votes (Calibration set)					NIR-PCs tree votes (Calibration set)				
		Class 1	Class 2	Class 3	Class 4	Class 5	Class 1	Class 2	Class 3	Class 4	Class 5
36	1	0.597931	0.164388	-0.01289	0.161221	0.089348	0.629326	0.113649	-0.11895	-0.19694	0.572913
33	1	0.86831	0.272421	0.160076	-0.06619	-0.23462	0.579341	0.319409	-0.03383	0.040364	0.094714
12	1	0.572817	0.101926	0.034199	0.391442	-0.10038	0.410747	-0.04919	0.3205	0.29664	0.021301
1	1	0.70159	0.152812	0.226027	-0.1565	0.076066	0.716141	-0.134	0.002468	0.349406	0.065988
2	1	1.04899	-0.22058	0.033554	0.041898	0.096143	0.335448	0.029993	-0.1003	-0.04374	0.778598
20	1	0.432027	0.163797	0.142196	0.15638	0.1056	0.7877	0.300961	0.235255	-0.02875	-0.29517
19	1	0.642368	-0.07632	0.220416	-0.05448	0.268016	0.803512	-0.43617	0.053685	0.229931	0.349043
27	1	0.543357	0.089069	0.148173	0.117285	0.102116	0.614847	0.061214	-0.01285	0.153177	0.183613
16	1	0.457262	0.142376	0.149428	0.047206	0.203728	0.732576	0.252152	-0.10843	-0.01059	0.134291
31	1	0.114985	-0.30488	0.569211	0.016236	0.604445	0.182161	0.124424	0.545251	0.001029	0.147136
3	1	0.939367	0.245811	0.048111	-0.07055	-0.16274	0.628124	-0.0104	-0.05113	0.149914	0.283491
18	1	0.71585	-0.37588	-0.14684	0.512762	0.29411	0.480893	0.116245	0.027305	0.350508	0.025048
9	1	0.666934	0.009583	-0.00335	0.241861	0.08497	0.829451	-0.08858	0.168761	0.032527	0.057841
26	1	0.621763	-0.10748	0.376221	0.023985	0.085506	0.732867	0.086351	0.115084	-0.0093	0.075003
25	1	1.13315	0.148482	-0.12797	-0.03109	-0.12257	0.290247	0.312298	-0.05334	0.14669	0.304106
23	1	0.473342	0.158171	0.109112	0.244719	0.014656	0.715474	0.060051	0.054279	-0.11653	0.28673
38	1	0.326513	0.233704	0.415762	-0.15616	0.180181	0.527818	-0.02694	0.034819	0.321358	0.14294
14	1	0.844055	-0.06526	0.167409	-0.04463	0.098424	0.552632	0.219746	0.437696	0.193293	-0.40337
40	1	0.214108	-0.07792	0.199447	0.122147	0.542214	0.512405	0.46573	-0.1935	0.253765	-0.0384
5	1	0.701396	0.169225	0.050295	0.126061	-0.04698	0.835764	-0.07283	-0.15667	0.438544	-0.0448
29	1	0.329659	0.33532	0.247156	-0.00855	0.096417	0.371546	-0.16241	0.165913	0.205902	0.419052
39	1	0.765143	0.145102	0.422831	-0.08019	-0.25289	0.753105	-0.11986	-0.28929	0.419082	0.236966
17	1	0.552698	0.20592	0.41082	-0.01752	-0.15192	0.415003	0.041352	0.125603	0.4379	-0.01986
7	1	0.621094	0.147709	0.112874	-0.09805	0.216377	0.549058	0.046229	0.143077	0.166253	0.095382
21	1	0.675329	0.302886	-0.3228	0.320622	0.023958	0.187454	0.368112	0.305157	0.225814	-0.08654
11	1	0.530895	0.144515	0.099913	0.006982	0.217695	0.51874	-0.22488	0.238678	0.15008	0.317381
62	2	0.103691	0.606705	0.519399	-0.09033	-0.13946	0.112026	1.00851	-0.27127	-0.01141	0.162139
59	2	0.041449	1.29232	-0.23302	0.243807	-0.34456	0.186184	0.639147	0.051864	0.132733	-0.00993
56	2	-0.61774	1.35864	0.394465	-0.02648	-0.10888	0.022471	0.909046	-0.0234	0.084327	0.007558
72	2	0.020596	1.11211	-0.31749	-0.10157	0.286358	0.577356	0.521325	-0.05425	0.089252	-0.13368
44	2	-0.07303	0.769707	0.076266	0.018561	0.208494	-0.03576	1.25563	0.053314	-0.21589	-0.0573
45	2	-0.15178	0.909009	-0.18514	0.074591	0.353325	0.083358	0.552356	0.257765	-0.27244	0.378965
63	2	0.614547	0.413158	0.39317	0.117801	-0.53868	0.036214	0.446779	0.240963	0.27307	0.002974
41	2	0.023296	0.405705	0.15309	0.272848	0.145061	-0.25885	0.784801	-0.03246	0.465835	0.040674
51	2	0.095554	0.372138	0.387691	-0.02453	0.169147	-0.12485	1.06072	-0.02857	0.250334	-0.15764
79	2	0.555813	0.675978	-0.27912	0.08077	-0.03344	-0.18306	0.938955	0.200218	0.077028	-0.03314
48	2	0.181706	0.562418	0.071366	0.169078	0.015433	0.043228	0.869443	0.140387	0.151483	-0.20454
76	2	0.412201	0.187349	0.361866	0.089417	-0.05083	0.133921	0.622326	0.201883	-0.10242	0.144289
68	2	-0.03654	1.06801	-0.21884	-0.05164	0.239017	-0.30591	1.04693	-0.14389	0.332451	0.070408
55	2	-0.26147	0.899493	0.32405	0.051517	-0.01359	0.300065	0.802686	-0.00965	-0.00248	-0.09062
61	2	0.121201	0.336353	0.123272	0.153323	0.265852	0.047562	0.897036	-0.18201	0.156474	0.080935
74	2	-0.07731	1.0315	0.025874	-0.08888	0.108815	-0.13131	1.15902	-0.25711	0.077739	0.151662
70	2	0.115256	0.48273	-0.02033	0.276524	0.145816	0.789435	0.486174	-0.08549	0.045631	-0.23575
54	2	-0.09343	0.79807	-0.05542	0.204654	0.146127	0.414727	0.456267	0.29097	-0.03888	-0.12309
66	2	0.073977	0.859089	0.006224	0.073306	-0.0126	0.100597	0.592077	0.074623	0.051622	0.181081
64	2	0.194152	0.491033	0.10146	0.154434	0.05892	-0.05844	0.631842	0.137394	0.161458	0.127746

57	2	0.313809	0.670634	0.048607	-0.05087	0.017817	-0.07358	0.847932	0.302647	-0.04947	-0.02753
75	2	0.17275	0.606984	-0.21428	0.049868	0.384672	0.298971	0.661393	-0.06781	0.080642	0.026804
47	2	0.358635	0.302903	0.154394	0.093269	0.0908	0.05444	0.929136	-0.11014	0.077657	0.048911
77	2	-0.07968	0.705489	0.351081	-0.18872	0.211835	-0.26978	0.785171	0.302508	0.036926	0.145179
53	2	0.270683	0.509466	0.051035	0.033844	0.134972	-0.12112	0.729061	0.066369	0.193583	0.132103
50	2	-0.17343	0.529882	0.27834	0.134289	0.230921	-0.04525	0.747346	0.248571	-0.15666	0.205988
101	3	0.304068	-0.30876	0.829932	0.067448	0.107315	-0.34233	-0.18454	1.15936	0.383486	-0.01598
91	3	0.312139	0.335089	0.551572	-0.16568	-0.03312	0.200636	0.004628	0.616141	0.132032	0.046563
90	3	0.231811	0.215235	0.633421	0.099639	-0.18011	0.273707	-0.06735	0.621989	0.049274	0.122383
87	3	0.389706	0.044699	0.475173	0.161257	-0.07084	-0.0827	0.036354	0.475297	0.345933	0.225119
99	3	-0.04572	0.20626	0.261252	0.218615	0.35959	0.215159	0.450654	0.421526	0.034302	-0.12164
114	3	0.2116	0.261575	0.29695	0.257264	-0.02739	-0.03566	-0.18722	1.01229	0.391636	-0.18105
116	3	0.340575	0.220081	0.357376	0.329191	-0.24722	-0.00469	-0.22412	0.794243	0.230967	0.203606
102	3	-0.06404	-0.31194	0.727562	0.269471	0.378948	0.046438	0.12177	0.623265	0.150328	0.0582
104	3	0.188116	0.150905	0.565705	-0.10173	0.197004	0.048202	0.020584	1.1792	-0.29045	0.042466
83	3	0.279599	0.135423	0.593482	-0.07587	0.067367	0.124241	0.180048	0.599054	0.024325	0.072332
110	3	0.038836	0.193956	0.394538	0.021912	0.350758	0.160332	0.022919	0.73405	0.01758	0.06512
108	3	0.338913	-0.03964	0.407012	-0.07072	0.364437	-0.01026	0.045448	1.06763	0.059102	-0.16192
81	3	-0.04094	0.079015	0.643693	0.281115	0.037119	0.344364	0.03856	0.776904	-0.06084	-0.09899
107	3	-0.00588	0.138796	0.17447	-0.04829	0.740906	0.234755	-0.01384	0.932104	0.041972	-0.19499
112	3	0.301787	-0.01263	0.415397	-0.09713	0.392575	0.118205	0.174433	0.807519	0.0158	-0.11596
113	3	0.174963	0.133787	0.375498	0.06633	0.249422	0.038373	0.224671	0.449967	0.266302	0.020686
97	3	0.156073	0.231393	0.552628	0.09254	-0.03263	0.1646	0.259188	0.655404	-0.0961	0.016907
85	3	0.086218	0.013026	0.484573	0.34842	0.067763	0.31413	0.078795	0.788455	0.003389	-0.18477
109	3	-0.02709	0.123825	0.326619	0.248693	0.327949	0.057488	0.14655	0.579773	0.122592	0.093599
105	3	-0.06142	0.010319	0.412994	0.219476	0.418626	-0.04041	0.010086	0.963305	0.034993	0.032024
100	3	0.252751	0.092081	0.351381	0.144862	0.158925	-0.06175	0.06709	0.82107	0.116931	0.056658
111	3	0.379739	0.123235	0.44551	-0.06719	0.118703	0.1696	-0.06027	0.695583	-0.02662	0.221702
96	3	-0.30572	-0.0402	0.40731	0.54195	0.396662	-0.03459	0.093274	0.694841	0.22674	0.019734
106	3	0.293299	0.302482	0.442362	0.019424	-0.05757	-0.04259	0.06572	0.820906	0.001892	0.154072
152	4	0.073186	0.020554	0.288142	0.541577	0.076542	0.273314	0.331002	0.213905	0.411975	-0.2302
121	4	0.011994	-0.15397	-0.03448	1.1502	0.026259	0.145914	-0.01511	0.074093	0.787902	0.007199
125	4	-0.04281	-0.25763	-0.06702	1.06834	0.299126	-0.11683	0.067847	0.026254	1.09033	-0.0676
128	4	-0.05115	-0.08939	-0.25263	1.42659	-0.03343	0.28408	-0.13794	0.304602	0.442232	0.107025
145	4	-0.06312	0.199692	0.067601	1.06889	-0.27305	-0.01032	0.0183	0.014919	1.07039	-0.09329
156	4	0.120301	-0.12325	0.291068	0.775799	-0.06392	0.439136	0.194802	-0.10743	0.600643	-0.12715
126	4	0.219146	0.043436	0.129754	0.753694	-0.14603	-0.23937	0.240876	-0.00227	0.788503	0.212256
131	4	0.200602	0.281689	-0.08851	0.783848	-0.17763	0.527318	-0.01672	7.67E-05	0.647148	-0.15782
117	4	0.180148	0.230828	0.02476	0.356876	0.207388	-0.26221	-0.23431	0.272161	0.97374	0.250618
122	4	0.020187	0.06892	0.232172	0.522172	0.156549	0.226879	0.01435	0.049204	0.442647	0.26692
149	4	0.272754	0.020376	0.098412	0.629756	-0.0213	0.192002	0.015746	0.330461	0.588606	-0.12682
153	4	0.069084	0.193544	0.028956	0.427775	0.280642	-0.07237	-0.13764	-0.00023	1.06375	0.146489
146	4	0.237058	-0.07075	0.043573	0.483547	0.306575	0.203301	0.008411	0.139033	0.655326	-0.00607
123	4	-0.02919	0.11696	0.008958	0.928362	-0.02509	0.235272	0.024333	0.140535	0.560502	0.039358
139	4	0.027797	0.020153	-0.04032	0.965166	0.027204	0.169704	0.197536	-0.09229	0.486448	0.238605
140	4	0.11626	0.041187	0.088046	0.625472	0.129035	0.107508	-0.1054	0.055032	1.00982	-0.06696
137	4	-0.01057	0.205609	0.399568	0.242153	0.163237	0.245164	0.300276	0.225973	0.274814	-0.04623
144	4	0.177154	0.171424	0.105599	0.272758	0.273064	0.271303	0.236086	0.136538	0.342253	0.01382
130	4	0.057383	0.143664	0.08647	0.864286	-0.1518	0.325262	0.217725	0.046569	0.315095	0.095348

118	4	0.021859	0.165215	0.184612	0.195823	0.432491	0.374222	-0.01746	-0.36652	1.06246	-0.05269
148	4	0.302596	0.050456	0.228985	0.44679	-0.02883	0.373895	0.142443	0.045869	0.373071	0.064723
134	4	-0.2757	-0.0408	0.432173	0.984047	-0.09972	-0.08752	0.059961	0.158789	0.640794	0.227979
141	4	0.052681	0.209003	0.01913	0.427085	0.292101	0.014597	0.035519	0.011871	0.784898	0.153116
136	4	0.040367	0.070593	0.149942	0.791673	-0.05257	-0.14663	0.163876	0.201432	0.840148	-0.05883
154	4	-0.09551	0.065574	0.249192	0.513177	0.267565	0.495562	-0.13478	0.183118	0.606581	-0.15048
129	4	0.114396	0.085786	0.086835	0.391765	0.321219	0.187131	0.41888	0.113881	0.234487	0.04562
192	5	0.105974	0.21009	0.123855	0.035483	0.524597	0.324229	-0.06989	-0.05389	-0.05452	0.854076
171	5	-0.01492	-0.17996	0.164204	0.16578	0.864893	0.022715	0.426374	-0.22078	0.040803	0.730891
169	5	0.168724	0.214974	-0.16847	0.201774	0.582993	0.068817	-0.00239	0.066901	0.416297	0.450375
162	5	-0.19447	0.177066	0.287113	0.157579	0.572708	0.07196	0.276179	0.373358	-0.39729	0.675789
182	5	0.383366	0.146621	-0.18475	-0.06348	0.718244	0.225322	-0.12442	-0.0991	0.239143	0.759054
190	5	-0.04333	0.269504	0.203401	0.287112	0.283311	0.153574	-0.00633	0.089794	-0.12213	0.885089
167	5	0.259003	-0.02376	0.495774	-0.11565	0.384628	0.45412	0.074541	-0.02906	0.040915	0.459489
178	5	0.216956	-0.11627	0.162537	-0.04271	0.779486	0.153435	0.241154	-0.19317	0.125628	0.672954
196	5	0.079427	-0.01097	-0.05604	0.20681	0.780768	0.315353	-0.02928	-0.04618	-0.02447	0.784579
165	5	0.078713	-0.16178	0.2597	0.446882	0.376484	0.315467	-0.14357	0.18051	-0.06282	0.710419
187	5	0.114424	0.296935	0.2529	-0.13302	0.468758	0.018637	0.162408	0.104353	0.005318	0.709284
191	5	-0.1397	-0.09728	0.462027	0.160716	0.614228	-0.11451	-0.31068	0.158645	-0.00629	1.27284
184	5	-0.06252	0.253851	0.15568	0.2433	0.40969	-0.25285	0.157682	0.262791	-0.18737	1.01974
161	5	-0.21152	0.354774	0.137756	0.074843	0.64415	0.099665	-0.11008	0.161871	0.029266	0.819277
180	5	0.187055	0.005876	0.215989	0.087011	0.504069	-0.00874	0.148521	-0.18953	0.109247	0.940503
175	5	-0.29862	0.464767	0.208889	0.184707	0.440258	0.291333	-0.12401	0.178556	-0.22005	0.874165
179	5	0.209983	-0.07119	0.185997	-0.11744	0.79265	0.240163	0.005967	-0.09508	0.307189	0.541765
168	5	0.412163	-0.02121	-0.05816	-0.06707	0.734284	0.170022	0.139036	-0.0364	-0.15582	0.883153
189	5	0.404423	0.060669	-0.084	-0.36589	0.984798	0.197291	-0.05983	-0.42079	0.544867	0.738462
173	5	0.149326	0.056989	0.117838	-0.00188	0.677731	0.133037	-0.12775	0.097069	0.149847	0.747793
194	5	0.068689	0.077621	0.517278	0.038248	0.298164	0.213898	0.054909	0.054327	0.166971	0.509895
195	5	0.123621	0.101637	0.167993	0.116788	0.489961	0.027073	0.080444	-0.00557	-0.15596	1.05401
170	5	0.021328	-0.07078	0.054999	0.205121	0.789335	0.081361	0.190756	-0.02478	0.017925	0.734742
174	5	0.072791	0.100366	0.093928	0.205076	0.527839	-0.01939	0.047672	0.160985	-0.04648	0.857213
163	5	0.147986	0.045893	0.069147	0.178848	0.558126	0.476254	-0.18554	0.003899	0.064842	0.640548
181	5	0.088996	-0.11309	0.299607	0.096669	0.62782	0.045173	0.122322	-0.10285	-0.14013	1.07548

Table S5

Sample number	Class label	FT-MIR-PCs tree votes (Validation set)					NIR-PCs tree votes (Validation set)				
		Class 1	Class 2	Class 3	Class 4	Class 5	Class 1	Class 2	Class 3	Class 4	Class 5
4	1	0.556318	0.214588	-0.00284	0.18775	0.044186	0.336733	0.060922	0.257577	0.045102	0.299667
6	1	0.345489	0.026074	0.07687	0.314535	0.237032	0.390643	0.038926	0.153193	0.514327	-0.09709
8	1	0.64447	0.159397	-0.24348	0.30763	0.13198	0.796322	-0.1688	0.183041	0.128509	0.060924
10	1	0.557143	0.042503	0.084181	0.12049	0.195683	0.486003	-0.14801	0.232206	0.184113	0.245692
13	1	0.472519	-0.08798	0.198399	0.197858	0.219208	0.462042	-0.23317	0.035837	0.425839	0.309451
15	1	0.550023	-0.06615	0.045254	0.412062	0.058812	0.474072	0.251561	0.38327	0.072015	-0.18092
22	1	0.399308	0.084315	0.019647	0.234918	0.261812	0.472007	0.119139	0.161046	0.120483	0.127326
24	1	0.285141	0.170383	0.109369	0.246926	0.188181	0.489641	0.099926	0.143729	0.207078	0.059626
28	1	0.241431	0.089347	0.297309	0.150271	0.221642	0.81034	-0.09729	0.137649	0.002503	0.146798
30	1	0.879942	0.128428	0.356793	-0.14822	-0.21694	0.767284	0.071131	0.179251	-0.03553	0.017861
32	1	0.742422	0.290095	0.148222	-0.13795	-0.04279	0.602853	0.132809	-0.08061	0.001104	0.343844
34	1	0.517709	-0.00813	0.195986	0.009069	0.285362	0.865393	-0.21393	0.397831	0.085481	-0.13477
35	1	0.388407	-0.041	0.423362	-0.05035	0.279581	0.484938	0.080371	-0.24888	0.34085	0.342719
37	1	0.496167	0.15004	0.364535	0.001946	-0.01269	0.750747	-0.08523	-0.01036	0.211371	0.133472
42	2	0.123822	0.44338	0.118988	0.142577	0.171235	0.418317	0.327362	0.007809	0.209094	0.037419
43	2	0.057731	0.457544	0.102578	0.251987	0.13016	0.41772	0.304946	0.076254	0.254663	-0.05358
46	2	0.042326	0.586553	-0.01673	0.150467	0.237388	0.259616	0.363906	-0.06286	0.149928	0.289413
49	2	0.181345	0.606676	0.205004	0.007477	-0.0005	0.282055	0.693737	-0.06391	0.182279	-0.09416
52	2	0.055452	0.501261	0.143565	0.12913	0.170593	0.238359	0.53498	0.015119	0.221014	-0.00947
58	2	0.080449	0.674165	0.090953	0.084855	0.069578	0.036267	0.683262	0.058285	-0.01334	0.235525
60	2	0.246211	0.658697	-0.19334	0.204197	0.084237	0.28025	0.619561	-0.10715	0.197944	0.009394
65	2	0.188806	0.513181	0.17897	-0.0196	0.138646	0.010651	0.896871	-0.25786	0.087667	0.262669
67	2	-0.05059	0.754613	0.095535	0.160646	0.039801	0.064844	0.5506	0.290648	0.39028	-0.29637
69	2	0.123692	0.524794	0.298485	-0.00037	0.053396	-0.09981	1.06515	0.180738	-0.15273	0.00664
71	2	-0.04426	0.534048	0.394476	-0.0419	0.157635	0.079716	0.928489	-0.05394	0.187462	-0.14172
73	2	0.019242	0.660525	0.101584	0.121229	0.09742	0.145657	0.660375	0.272644	-0.0921	0.013427
78	2	0.068094	0.739191	-0.08767	0.059738	0.220646	-0.17851	0.916661	0.176329	0.060597	0.024921
80	2	0.112094	0.419513	-0.02478	0.185029	0.308145	-0.05478	0.576168	-0.00725	0.035562	0.450296
82	3	0.257177	0.135249	0.44248	-0.00042	0.165516	0.25498	0.134327	0.445111	0.268506	-0.10292
84	3	0.136507	0.149636	0.432791	0.172499	0.108566	-0.17442	0.216385	0.949182	0.084149	-0.0753
86	3	0.220052	0.138735	0.437148	0.115193	0.088872	0.135369	-0.07204	0.604658	0.271334	0.060674
88	3	-0.06982	0.171922	0.279162	0.195019	0.423721	0.158954	0.001729	0.65633	0.084716	0.098271
89	3	-0.13472	0.183704	0.238779	0.272142	0.440091	0.134716	-0.06018	0.785965	0.05351	0.085985
92	3	0.239074	0.288794	0.425272	-0.02324	0.070102	0.208065	0.331145	0.59149	-0.182	0.051304
93	3	0.284963	0.16928	0.341851	0.134091	0.069815	-0.1008	0.031589	0.906255	-0.17265	0.33561
94	3	0.151631	0.152134	0.387887	0.22899	0.079358	0.28673	-0.00056	0.504722	-0.09813	0.307232
95	3	0.31709	0.278125	0.440652	-0.07169	0.035823	0.107984	0.006537	0.752368	0.017911	0.115201
98	3	-0.04451	0.32351	0.252888	0.240546	0.227569	0.347147	0.024359	0.533589	-0.09971	0.194613
103	3	-0.06962	0.069258	0.477546	0.332674	0.190142	-0.27979	0.142642	0.711013	0.274796	0.151336
115	3	0.055563	0.35888	0.412347	0.152884	0.020326	0.201608	-0.00845	0.764708	0.227156	-0.18502
119	4	-0.09216	0.212433	0.213688	0.94245	-0.27641	0.046241	0.229853	-0.02327	0.569103	0.178077
120	4	-0.08475	0.111964	0.064566	0.931184	-0.02296	0.151367	-0.02508	0.190301	0.591096	0.092312
124	4	0.045404	0.277649	0.106852	0.68299	-0.1129	0.425273	0.030904	0.146724	0.574649	-0.17755
127	4	-0.07499	0.279413	-0.08433	1.0589	-0.17899	0.160214	0.129721	0.414294	0.484116	-0.18835
132	4	-0.083	0.242329	0.05169	0.934001	-0.14502	0.596815	-0.05043	-0.02556	0.572522	-0.09334
133	4	0.101048	0.14792	-0.08647	0.83376	0.003743	0.119816	0.161369	0.151957	0.567357	-0.0005

135	4	0.005472	0.235083	0.075173	0.302055	0.382216	0.069047	0.097669	0.183404	0.516011	0.133869
138	4	-0.04733	0.218716	0.208353	0.404734	0.215526	0.199122	0.163549	0.288038	0.394456	-0.04516
142	4	0.061696	-0.00486	0.261805	0.531797	0.149566	0.023141	0.208705	0.068737	0.490489	0.208929
143	4	-0.05365	-0.04916	0.28764	0.578253	0.236922	0.1225	0.099443	0.555304	0.192175	0.030579
147	4	-0.02753	0.114521	0.10019	0.52898	0.283836	0.11759	0.057658	0.208331	0.391678	0.224742
150	4	0.197817	0.002403	0.14184	0.439556	0.218385	0.328323	-0.02727	0.176323	0.546402	-0.02378
151	4	-0.03436	0.130725	0.176951	0.531251	0.19543	0.268457	0.02086	-0.06045	0.531746	0.239383
155	4	-0.07592	0.194658	0.364302	0.34591	0.171048	0.163938	0.22711	0.019221	0.273322	0.31641
157	5	0.071081	-0.10458	0.138297	0.163488	0.731716	0.175873	0.144691	-0.07857	0.177422	0.580583
158	5	-0.01434	0.003875	0.251583	0.202397	0.556487	0.336861	-0.08054	0.15333	0.276561	0.313793
159	5	0.163042	0.095325	0.038296	0.197456	0.505881	0.204673	0.189022	-0.07226	0.196459	0.482102
160	5	0.001074	-0.08563	0.13277	0.36083	0.590956	0.456186	-0.01022	-0.12682	0.107219	0.573638
164	5	0.034998	0.003172	0.155464	0.361848	0.444518	0.178191	-0.0483	0.06519	0.234098	0.570816
166	5	0.001045	-0.03279	0.309678	0.016584	0.705481	0.226693	0.093414	-0.12966	0.246743	0.562811
172	5	0.1109	0.052248	0.215498	0.134511	0.486844	0.486445	-0.20266	0.085788	0.119021	0.511404
176	5	0.031761	0.018122	0.244769	0.221951	0.483397	0.360301	0.092742	-0.28844	0.140027	0.695366
177	5	0.229924	0.124458	0.179607	-0.07894	0.544953	0.046675	0.106631	0.128897	-0.13267	0.850464
183	5	0.078339	0.070088	0.145353	0.043102	0.663118	0.065284	0.069762	0.086698	-0.08068	0.858938
185	5	0.208755	-0.14639	0.18296	-0.16296	0.917643	0.276303	0.176467	0.053952	-0.07504	0.568321
186	5	0.079643	0.058651	0.14349	0.065512	0.652704	0.023116	-0.064	0.263001	-0.24402	1.02191
188	5	0.089812	-0.04397	0.309688	0.099405	0.545065	0.330447	0.004715	0.04323	0.075962	0.545646
193	5	-0.14245	0.178626	0.011237	0.123821	0.828762	-0.10261	0.091638	-0.08605	-0.16746	1.26448

Table S6

Sample number	Class label	FT-MIR-RFE tree votes (Calibration set)					NIR-RFE tree votes (Calibration set)				
		Class 1	Class 2	Class 3	Class 4	Class 5	Class 1	Class 2	Class 3	Class 4	Class 5
36	1	0.428571	0.125	0.089286	0.089286	0.267857	0.356757	0.084685	0.091892	0.190991	0.275676
33	1	0.798077	0.048077	0.076923	0	0.076923	0.360656	0.083789	0.091075	0.052823	0.411658
12	1	0.41	0.23	0.25	0.07	0.04	0.287449	0.121457	0.198381	0.143725	0.248988
1	1	0.5	0.064815	0.222222	0.166667	0.046296	0.432971	0.023551	0.099638	0.036232	0.407609
2	1	0.09901	0.158416	0.405941	0.277228	0.059406	0.401099	0.029304	0.06044	0.069597	0.43956
20	1	0.582524	0.048544	0.019417	0.029126	0.320388	0.217726	0.22158	0.235067	0.256262	0.069364
19	1	0.654867	0.061947	0.221239	0.035398	0.026549	0.19337	0.414365	0.191529	0.145488	0.055249
27	1	0.943925	0.009346	0.018692	0.009346	0.018692	0.331481	0.124074	0.124074	0.092593	0.327778
16	1	0.537736	0.028302	0.04717	0.028302	0.358491	0.191095	0.168831	0.231911	0.376623	0.03154
31	1	0.336449	0.224299	0.018692	0.130841	0.28972	0.414855	0.067029	0.048913	0.072464	0.396739
3	1	0.547368	0.115789	0.210526	0.063158	0.063158	0.524254	0.08209	0.074627	0.039179	0.279851
18	1	0.779817	0.055046	0.018349	0.055046	0.091743	0.282609	0.313406	0.231884	0.139493	0.032609
9	1	0.847619	0.085714	0.019048	0.019048	0.028571	0.495528	0.053667	0.173524	0.033989	0.243292
26	1	0.54902	0.205882	0.068627	0.019608	0.156863	0.630769	0.086538	0.153846	0.032692	0.096154
25	1	0.376	0.144	0.048	0.184	0.248	0.423529	0.060784	0.2	0.139216	0.176471
23	1	0.352941	0.107843	0.147059	0.235294	0.156863	0.245779	0.140713	0.187617	0.223265	0.202627
38	1	0.55102	0.255102	0.030612	0.061224	0.102041	0.549645	0.090426	0.124113	0.04078	0.195035
14	1	0.543689	0.07767	0.067961	0.126214	0.184466	0.195029	0.231358	0.231358	0.33652	0.005736
40	1	0.424779	0.265487	0.141593	0.053097	0.115044	0.376448	0.073359	0.083012	0.061776	0.405405
5	1	0.793814	0.082474	0.061856	0.020619	0.041237	0.283662	0.104129	0.195691	0.344704	0.071813
29	1	0.907407	0.027778	0.027778	0	0.037037	0.662879	0.104167	0.100379	0.034091	0.098485
39	1	0.810811	0.045045	0.036036	0.045045	0.063063	0.528155	0.126214	0.099029	0.087379	0.159223
17	1	0.523364	0.028037	0.084112	0.037383	0.327103	0.23327	0.321224	0.217973	0.128107	0.099426
7	1	0.757282	0.126214	0.087379	0.019417	0.009709	0.454365	0.134921	0.186508	0.107143	0.117063
21	1	0.18018	0.081081	0.252252	0.27027	0.216216	0.177388	0.031189	0.037037	0.060429	0.693957
11	1	0.822222	0.066667	0.055556	0.011111	0.044444	0.449438	0.031835	0.117978	0.104869	0.29588
62	2	0.035088	0.719298	0.210526	0.026316	0.008772	0.098456	0.73166	0.125483	0.034749	0.009653
59	2	0.23301	0.398058	0.174757	0.07767	0.116505	0.108159	0.609108	0.163188	0.096774	0.02277
56	2	0.103448	0.353448	0.206897	0.103448	0.232759	0.168856	0.559099	0.146341	0.099437	0.026266
72	2	0.067797	0.610169	0.186441	0.042373	0.09322	0.176147	0.581651	0.07156	0.097248	0.073394
44	2	0.034884	0.5	0.383721	0.034884	0.046512	0.146691	0.531306	0.152057	0.096601	0.073345
45	2	0.201923	0.384615	0.259615	0.067308	0.086538	0.132296	0.505837	0.237354	0.087549	0.036965
63	2	0.069307	0.445545	0.128713	0.237624	0.118812	0.1171	0.457249	0.208178	0.152416	0.065056
41	2	0.12605	0.05042	0.10084	0.201681	0.521008	0.184502	0.151292	0.132841	0.494465	0.0369
51	2	0.214953	0.514019	0.205607	0.018692	0.046729	0.302326	0.412791	0.160853	0.036822	0.087209
79	2	0.067961	0.61165	0.135922	0.038835	0.145631	0.117537	0.735075	0.052239	0.048507	0.046642
48	2	0.111111	0.648148	0.092593	0.046296	0.101852	0.186898	0.55684	0.073218	0.142582	0.040462
76	2	0.091743	0.541284	0.174312	0.036697	0.155963	0.188119	0.584158	0.120792	0.055446	0.051485
68	2	0.19469	0.256637	0.168142	0.327434	0.053097	0.185687	0.441006	0.305609	0.058027	0.009671
55	2	0.343434	0.353535	0.131313	0.070707	0.10101	0.159785	0.581688	0.184919	0.050269	0.023339
61	2	0.046512	0.5	0.116279	0.27907	0.05814	0.09369	0.351816	0.263862	0.258126	0.032505
74	2	0.230769	0.519231	0.115385	0.028846	0.105769	0.094303	0.691552	0.143418	0.041257	0.02947
70	2	0	0.573913	0.113043	0.26087	0.052174	0.08958	0.316271	0.129799	0.420475	0.043876
54	2	0.191304	0.417391	0.286957	0.06087	0.043478	0.252372	0.478178	0.1537	0.060721	0.055028
66	2	0.044248	0.778761	0.088496	0.026549	0.061947	0.088015	0.659176	0.153558	0.052434	0.046816
64	2	0.031579	0.621053	0.147368	0.168421	0.031579	0.157801	0.203901	0.200355	0.391844	0.046099

57	2	0.029126	0.757282	0.07767	0.038835	0.097087	0.070881	0.62069	0.118774	0.159004	0.030651
75	2	0.192308	0.538462	0.067308	0.096154	0.105769	0.117534	0.662813	0.154143	0.032755	0.032755
47	2	0.066038	0.537736	0.301887	0.028302	0.066038	0.173358	0.538321	0.136861	0.105839	0.04562
77	2	0.037037	0.444444	0.138889	0.074074	0.305556	0.190741	0.42963	0.212963	0.087037	0.07963
53	2	0.138298	0.404255	0.138298	0.095745	0.223404	0.20075	0.36773	0.247655	0.140713	0.043152
50	2	0.02	0.76	0.13	0.04	0.05	0.139623	0.271698	0.132075	0.375472	0.081132
101	3	0.142857	0.071429	0.295918	0.326531	0.163265	0.078	0.176	0.5	0.192	0.054
91	3	0.144444	0.144444	0.522222	0.011111	0.177778	0.365714	0.24	0.16	0.08	0.154286
90	3	0.036036	0.207207	0.279279	0.279279	0.198198	0.049713	0.053537	0.156788	0.476099	0.263862
87	3	0.27193	0.175439	0.438596	0.035088	0.078947	0.339318	0.240575	0.18851	0.071813	0.159785
99	3	0.09434	0.264151	0.566038	0.04717	0.028302	0.132353	0.481618	0.220588	0.150735	0.014706
114	3	0.057143	0.485714	0.333333	0.038095	0.085714	0.193424	0.065764	0.510638	0.077369	0.152805
116	3	0.065421	0.158879	0.626168	0.121495	0.028037	0.15625	0.0625	0.617647	0.073529	0.090074
102	3	0.091743	0.055046	0.275229	0.440367	0.137615	0.068783	0.137566	0.40388	0.35097	0.038801
104	3	0.232323	0.151515	0.484848	0.090909	0.040404	0.137405	0.093511	0.59542	0.116412	0.057252
83	3	0.228571	0.057143	0.619048	0.038095	0.057143	0.230909	0.376364	0.290909	0.069091	0.032727
110	3	0.091837	0.22449	0.408163	0.132653	0.142857	0.094096	0.086716	0.573801	0.208487	0.0369
108	3	0.114286	0.180952	0.257143	0.066667	0.380952	0.175652	0.074783	0.56	0.111304	0.078261
81	3	0.066667	0.057143	0.552381	0.152381	0.171429	0.181004	0.263441	0.299283	0.166667	0.089606
107	3	0.131579	0.035088	0.5	0.27193	0.061404	0.10397	0.126654	0.466919	0.238185	0.064272
112	3	0.009901	0.207921	0.534653	0.227723	0.019802	0.133333	0.04381	0.535238	0.139048	0.148571
113	3	0.046729	0.280374	0.598131	0.056075	0.018692	0.354167	0.11553	0.371212	0.068182	0.090909
97	3	0.019608	0.058824	0.401961	0.480392	0.039216	0.099237	0.076336	0.221374	0.55916	0.043893
85	3	0.067308	0.153846	0.519231	0.163462	0.096154	0.266284	0.266284	0.268199	0.172414	0.02682
109	3	0.310345	0.172414	0.189655	0.137931	0.189655	0.173835	0.071685	0.523297	0.134409	0.096774
105	3	0	0.095745	0.56383	0.244681	0.095745	0.052838	0.050881	0.459883	0.330724	0.105675
100	3	0.045872	0.137615	0.541284	0.211009	0.06422	0.143925	0.24486	0.285981	0.304673	0.020561
111	3	0.037736	0.141509	0.632075	0.141509	0.04717	0.071563	0.043315	0.45951	0.333333	0.092279
96	3	0.145455	0.381818	0.381818	0.018182	0.072727	0.180952	0.466667	0.211429	0.097143	0.04381
106	3	0.094828	0.163793	0.560345	0.137931	0.043103	0.316505	0.132039	0.266019	0.085437	0.2
152	4	0.141667	0.208333	0.416667	0.116667	0.116667	0.21371	0.324597	0.1875	0.233871	0.040323
121	4	0.204918	0.040984	0.090164	0.581967	0.081967	0.096774	0.125237	0.079696	0.633776	0.064516
125	4	0	0.136752	0.102564	0.726496	0.034188	0.02037	0.055556	0.196296	0.651852	0.075926
128	4	0.009009	0.054054	0.081081	0.81982	0.036036	0.105751	0.040816	0.176252	0.58256	0.09462
145	4	0.115789	0.084211	0.326316	0.336842	0.136842	0.326886	0.158607	0.2147	0.17795	0.121857
156	4	0.091837	0.112245	0.234694	0.489796	0.071429	0.190741	0.174074	0.162963	0.387037	0.085185
126	4	0	0.116667	0.066667	0.816667	0	0.134122	0.213018	0.120316	0.319527	0.213018
131	4	0.050847	0.067797	0.067797	0.788136	0.025424	0.227437	0.274368	0.122744	0.310469	0.064982
117	4	0.018692	0.028037	0.084112	0.859813	0.009346	0.060714	0.15	0.135714	0.632143	0.021429
122	4	0.059406	0.049505	0.089109	0.782178	0.019802	0.083176	0.151229	0.10586	0.610586	0.049149
149	4	0.074074	0.037037	0.111111	0.506173	0.271605	0.203738	0.031776	0.22243	0.276636	0.265421
153	4	0.148148	0.092593	0.25	0.333333	0.175926	0.13945	0.111927	0.315596	0.366972	0.066055
146	4	0.015385	0.007692	0.038462	0.738462	0.2	0.272388	0.070896	0.147388	0.330224	0.179104
123	4	0.044643	0.107143	0.053571	0.785714	0.008929	0.275194	0.22093	0.232558	0.149225	0.122093
139	4	0.179245	0.04717	0.320755	0.320755	0.132075	0.126437	0.132184	0.249042	0.454023	0.038314
140	4	0.028571	0.152381	0.104762	0.419048	0.295238	0.037106	0.046382	0.122449	0.755102	0.038961
137	4	0.096491	0.192982	0.184211	0.219298	0.307018	0.066148	0.081712	0.245136	0.570039	0.036965
144	4	0.465517	0.155172	0.060345	0.094828	0.224138	0.225	0.110714	0.289286	0.291071	0.083929
130	4	0	0.035714	0.080357	0.866071	0.017857	0.063636	0.058182	0.234545	0.578182	0.065455

118	4	0.028302	0.084906	0.113208	0.754717	0.018868	0.131579	0.145614	0.15614	0.526316	0.040351
148	4	0.028571	0.047619	0.057143	0.733333	0.133333	0.082061	0.026718	0.166031	0.498092	0.227099
134	4	0.040404	0.131313	0.151515	0.181818	0.494949	0.085657	0.071713	0.145418	0.589641	0.10757
141	4	0.016393	0.057377	0.172131	0.598361	0.155738	0.112186	0.079304	0.237911	0.516441	0.054159
136	4	0.05	0.11	0.23	0.31	0.3	0.051786	0.0375	0.130357	0.7375	0.042857
154	4	0.047619	0.057143	0.257143	0.590476	0.047619	0.201117	0.173184	0.176909	0.350093	0.098696
129	4	0.017857	0.044643	0	0.919643	0.017857	0.080645	0.102151	0.16129	0.455197	0.200717
192	5	0.115702	0.256198	0.165289	0.140496	0.322314	0.225379	0.058712	0.05303	0.041667	0.621212
171	5	0.045872	0.045872	0.137615	0.376147	0.394495	0.09073	0.019724	0.084813	0.128205	0.676529
169	5	0.293578	0.082569	0.137615	0.165138	0.321101	0.17658	0.111524	0.091078	0.289963	0.330855
162	5	0.009259	0.055556	0.055556	0.12037	0.759259	0.041825	0.015209	0.18251	0.165399	0.595057
182	5	0.142857	0.142857	0.085714	0.057143	0.571429	0.636711	0.042065	0.036329	0.022945	0.26195
190	5	0.156863	0.186275	0.107843	0.098039	0.45098	0.274908	0.173432	0.197417	0.053506	0.300738
167	5	0.405172	0.051724	0.077586	0.025862	0.439655	0.215953	0.011673	0.038911	0.046693	0.68677
178	5	0.25	0.12963	0.222222	0.092593	0.305556	0.132379	0.042934	0.078712	0.046512	0.699463
196	5	0.223214	0.116071	0.107143	0.160714	0.392857	0.433892	0.029795	0.080074	0.011173	0.445065
165	5	0.030303	0.030303	0.111111	0.121212	0.707071	0.210238	0.025594	0.060329	0.122486	0.581353
187	5	0.093458	0.140187	0.102804	0.121495	0.542056	0.250951	0.068441	0.153992	0.106464	0.420152
191	5	0.31068	0.145631	0.145631	0.165049	0.23301	0.357782	0.060823	0.087657	0.025045	0.468694
184	5	0.405405	0.126126	0.045045	0.018018	0.405405	0.251786	0.098214	0.133929	0.064286	0.451786
161	5	0.063063	0.072072	0.162162	0.18018	0.522523	0.098485	0.005682	0.140152	0.143939	0.611742
180	5	0.211009	0.348624	0.211009	0.036697	0.192661	0.325967	0.053407	0.053407	0.029466	0.537753
175	5	0.181818	0.050505	0.040404	0.090909	0.636364	0.114603	0.011091	0.075786	0.072089	0.726433
179	5	0.252632	0.084211	0.073684	0.052632	0.536842	0.194831	0.049702	0.111332	0.065606	0.578529
168	5	0.054054	0.036036	0.153153	0.117117	0.63964	0.258755	0.149805	0.070039	0.249027	0.272374
189	5	0.158879	0.130841	0.028037	0.037383	0.64486	0.499112	0.058615	0.101243	0.0373	0.30373
173	5	0.070796	0.053097	0.044248	0.061947	0.769912	0.152047	0.021442	0.074074	0.19883	0.553606
194	5	0.017391	0.286957	0.086957	0.226087	0.382609	0.089552	0.037313	0.106343	0.080224	0.686567
195	5	0.042735	0.239316	0.068376	0.136752	0.512821	0.179775	0.041199	0.052434	0.061798	0.664794
170	5	0.027778	0.064815	0.12963	0.175926	0.601852	0.109562	0.02988	0.11753	0.35259	0.390438
174	5	0.078431	0.107843	0.078431	0.156863	0.578431	0.092764	0.012987	0.048237	0.027829	0.818182
163	5	0.048077	0.048077	0.057692	0.057692	0.788462	0.096386	0.004016	0.054217	0.13253	0.712851
181	5	0.083333	0.212963	0.185185	0.055556	0.462963	0.246777	0.053407	0.066298	0.106814	0.526703

Table S7

Sample number	Class label	FT-MIR-RFE tree votes (Validation set)					NIR-RFE tree votes (Validation set)				
		Class 1	Class 2	Class 3	Class 4	Class 5	Class 1	Class 2	Class 3	Class 4	Class 5
4	1	0.587031	0.068259	0.273038	0.054608	0.017065	0.196699	0.228336	0.335626	0.21458	0.024759
6	1	0.293515	0.331058	0.146758	0.09215	0.136519	0.178129	0.083906	0.19326	0.484182	0.060523
8	1	0.457338	0.177474	0.116041	0.085324	0.163823	0.576341	0.07359	0.094911	0.041265	0.213893
10	1	0.853242	0.068259	0.027304	0.020478	0.030717	0.470426	0.02751	0.107978	0.028886	0.365199
13	1	0.450512	0.098976	0.133106	0.112628	0.204778	0.232462	0.264099	0.137552	0.322558	0.043329
15	1	0.109215	0.150171	0.52901	0.163823	0.047782	0.158872	0.162999	0.317056	0.277854	0.083219
22	1	0.327645	0.064846	0.047782	0.071672	0.488055	0.261348	0.132737	0.218707	0.249656	0.137552
24	1	0.539249	0.081911	0.040956	0.098976	0.238908	0.3989	0.059835	0.099037	0.232462	0.209766
28	1	0.737201	0.061433	0.071672	0.044369	0.085324	0.641678	0.053645	0.136176	0.068088	0.100413
30	1	0.723549	0.044369	0.030717	0.054608	0.146758	0.48762	0.055021	0.089409	0.076341	0.291609
32	1	0.324232	0.054608	0.16041	0.044369	0.416382	0.41403	0.041265	0.068776	0.112792	0.363136
34	1	0.426621	0.102389	0.09215	0.143345	0.235495	0.542641	0.033012	0.174691	0.038514	0.211142
35	1	0.453925	0.047782	0.167235	0.112628	0.21843	0.563274	0.024759	0.041265	0.108666	0.262036
37	1	0.590444	0.116041	0.071672	0.040956	0.180887	0.624484	0.0337	0.063961	0.029574	0.248281
42	2	0.105802	0.273038	0.286689	0.191126	0.143345	0.257221	0.310179	0.255158	0.14099	0.036451
43	2	0.061433	0.296928	0.313993	0.21843	0.109215	0.197387	0.321871	0.143054	0.277166	0.060523
46	2	0.030717	0.631399	0.180887	0.081911	0.075085	0.111417	0.218019	0.118294	0.474553	0.077717
49	2	0.054608	0.832765	0.061433	0.013652	0.037543	0.121045	0.600413	0.141678	0.072215	0.064649
52	2	0.020478	0.726962	0.116041	0.05802	0.078498	0.151994	0.387208	0.261348	0.126547	0.072902
58	2	0.098976	0.573379	0.064846	0.116041	0.146758	0.100413	0.396149	0.160935	0.321871	0.020633
60	2	0.027304	0.778157	0.112628	0.030717	0.051195	0.03989	0.860385	0.063274	0.022696	0.013755
65	2	0.010239	0.849829	0.088737	0.023891	0.027304	0.094911	0.657497	0.101788	0.11967	0.026135
67	2	0.020478	0.805461	0.078498	0.085324	0.010239	0.110041	0.590096	0.182944	0.060523	0.056396
69	2	0.023891	0.723549	0.136519	0.081911	0.03413	0.124484	0.562586	0.22077	0.059147	0.033012
71	2	0.040956	0.750853	0.088737	0.023891	0.095563	0.128611	0.724209	0.06121	0.042641	0.043329
73	2	0.054608	0.587031	0.1843	0.112628	0.061433	0.156809	0.570839	0.123109	0.122421	0.026823
78	2	0.020478	0.706485	0.139932	0.047782	0.085324	0.093535	0.727648	0.123796	0.026823	0.028198
80	2	0.020478	0.464164	0.249147	0.170648	0.095563	0.151994	0.555708	0.122421	0.06121	0.108666
82	3	0.102389	0.05802	0.750853	0.03413	0.054608	0.176754	0.313618	0.325309	0.156121	0.028198
84	3	0.064846	0.167235	0.610922	0.102389	0.054608	0.227648	0.175378	0.36795	0.14718	0.081843
86	3	0.109215	0.064846	0.648464	0.109215	0.068259	0.234525	0.216644	0.303301	0.105915	0.139615
88	3	0.013652	0.204778	0.47099	0.194539	0.116041	0.074966	0.088033	0.275103	0.491059	0.070839
89	3	0.030717	0.156997	0.477816	0.225256	0.109215	0.083906	0.060523	0.271664	0.470426	0.11348
92	3	0.078498	0.16041	0.658703	0.054608	0.047782	0.442916	0.124484	0.136864	0.019945	0.275791
93	3	0.027304	0.129693	0.74744	0.044369	0.051195	0.389959	0.110041	0.232462	0.057084	0.210454
94	3	0.051195	0.040956	0.723549	0.102389	0.081911	0.337689	0.058459	0.202889	0.101788	0.299175
95	3	0.307167	0.075085	0.501706	0.078498	0.037543	0.394773	0.078404	0.186382	0.041265	0.299175
98	3	0.027304	0.09215	0.447099	0.399317	0.03413	0.127923	0.103164	0.330124	0.403026	0.035763
103	3	0.030717	0.064846	0.549488	0.197952	0.156997	0.050894	0.057772	0.372765	0.473865	0.044704
115	3	0.05802	0.150171	0.682594	0.081911	0.027304	0.167125	0.082531	0.493122	0.07359	0.183631
119	4	0.006826	0.167235	0.061433	0.750853	0.013652	0.045392	0.078404	0.152682	0.691197	0.032325
120	4	0.006826	0.03413	0.047782	0.90785	0.003413	0.054333	0.044017	0.084594	0.788171	0.028886
124	4	0.003413	0.027304	0.010239	0.955631	0.003413	0.125172	0.090096	0.103164	0.599037	0.082531
127	4	0.003413	0.068259	0.051195	0.870307	0.006826	0.069464	0.05227	0.15337	0.665062	0.059835
132	4	0.013652	0.054608	0.03413	0.880546	0.017065	0.136176	0.136176	0.104539	0.577717	0.045392
133	4	0	0.013652	0.030717	0.955631	0	0.044704	0.050206	0.144429	0.743466	0.017194

135	4	0.078498	0.064846	0.153584	0.327645	0.375427	0.151307	0.131362	0.091472	0.546768	0.079092
138	4	0.013652	0.03413	0.09215	0.559727	0.300341	0.049519	0.055708	0.157497	0.682944	0.054333
142	4	0.054608	0.071672	0.102389	0.474403	0.296928	0.0674	0.068776	0.29436	0.51238	0.057084
143	4	0.013652	0.05802	0.075085	0.713311	0.139932	0.061898	0.092847	0.093535	0.695323	0.056396
147	4	0.020478	0.020478	0.075085	0.8157	0.068259	0.100413	0.054333	0.223521	0.517194	0.104539
150	4	0.023891	0.023891	0.068259	0.771331	0.112628	0.094223	0.050894	0.20564	0.470426	0.178817
151	4	0.006826	0.027304	0.273038	0.583618	0.109215	0.189133	0.030949	0.118294	0.297111	0.364512
155	4	0.03413	0.037543	0.211604	0.686007	0.030717	0.272352	0.182944	0.096286	0.389959	0.058459
157	5	0.020478	0.12628	0.153584	0.204778	0.494881	0.058459	0.029574	0.271664	0.354195	0.286107
158	5	0.010239	0.044369	0.054608	0.098976	0.791809	0.072215	0.023384	0.149931	0.352132	0.402338
159	5	0.017065	0.020478	0.044369	0.119454	0.798635	0.066713	0.022008	0.18088	0.338377	0.392022
160	5	0.030717	0.030717	0.040956	0.102389	0.795222	0.068088	0.013755	0.128611	0.226272	0.563274
164	5	0.020478	0.03413	0.071672	0.105802	0.767918	0.15956	0.011692	0.041265	0.088721	0.698762
166	5	0.05802	0.006826	0.020478	0.047782	0.866894	0.096974	0.011692	0.049519	0.171252	0.670564
172	5	0.020478	0.071672	0.095563	0.167235	0.645051	0.074278	0.011004	0.106602	0.163686	0.644429
176	5	0.170648	0.071672	0.088737	0.136519	0.532423	0.089409	0.008941	0.02132	0.03989	0.84044
177	5	0.129693	0.136519	0.450512	0.05802	0.225256	0.295048	0.040578	0.136176	0.04608	0.482118
183	5	0.279863	0.023891	0.047782	0.020478	0.627986	0.281981	0.110729	0.098349	0.051582	0.457359
185	5	0.051195	0.156997	0.139932	0.12628	0.525597	0.206327	0.044704	0.077029	0.06121	0.610729
186	5	0.201365	0.143345	0.122867	0.047782	0.484642	0.296424	0.070839	0.083906	0.048831	0.5
188	5	0.051195	0.047782	0.102389	0.225256	0.573379	0.107978	0.034388	0.091472	0.104539	0.661623
193	5	0.023891	0.197952	0.03413	0.081911	0.662116	0.081843	0.025447	0.055708	0.07359	0.763411

Table S8

Sample number	Class label	FT-MIR-RFE tree votes (Calibration set)					NIR-RFE tree votes (Calibration set)				
		Class 1	Class 2	Class 3	Class 4	Class 5	Class 1	Class 2	Class 3	Class 4	Class 5
36	1	0.37357	-0.01733	0.214296	0.149037	0.280423	0.664562	-0.09413	-0.21009	0.508672	0.130982
33	1	0.99893	0.226294	-0.14351	-0.10054	0.018824	0.659185	0.094052	-0.1692	0.094146	0.321821
12	1	0.488894	0.139241	0.283868	0.083371	0.004626	0.729267	0.040594	-0.20251	0.455871	-0.02322
1	1	0.860409	-0.07937	0.001514	0.285996	-0.06855	0.579864	-0.24267	0.150767	0.16643	0.345611
2	1	0.353264	0.333248	0.044408	0.177192	0.091888	0.590685	-0.20181	-0.02819	0.292453	0.346865
20	1	0.595524	-0.22427	0.333501	-0.22102	0.516264	0.27581	0.377358	0.113518	0.393474	-0.16016
19	1	0.777141	-0.11877	0.46603	-0.13905	0.014656	0.284103	0.387504	0.083537	0.354553	-0.1097
27	1	0.769404	0.147239	0.013618	0.017654	0.052085	0.633232	0.072581	0.027483	-0.00324	0.269941
16	1	0.191435	-0.20082	0.335811	-0.15524	0.828811	0.082116	0.221942	0.389894	0.39699	-0.09094
31	1	0.510965	0.217858	0.001777	0.211471	0.057929	0.595898	0.05993	-0.3199	0.357943	0.306128
3	1	0.843273	-0.04998	0.05138	0.04614	0.109191	0.670298	0.122116	-0.09285	0.046332	0.254103
18	1	0.774367	-0.09972	0.199454	-0.08389	0.209795	0.347515	0.175249	0.486316	0.141989	-0.15107
9	1	0.729525	0.073807	0.182592	0.031967	-0.01789	0.560541	5+J3:J2	0.223108	0.006353	0.260917
26	1	0.871553	0.045883	0.202423	-0.06731	-0.05255	0.684738	0.139071	-0.00274	0.128798	0.05013
25	1	0.33872	0.17975	0.062187	0.191195	0.228147	0.76954	-0.17285	0.048643	0.343005	0.011662
23	1	0.484014	0.281393	-0.09062	0.259072	0.066144	0.441507	0.162575	-0.08717	0.254079	0.229008
38	1	0.552461	0.240308	0.176762	0.211248	-0.18078	0.657873	0.11294	0.088815	0.045673	0.094698
14	1	0.763226	-0.43843	0.215265	0.236523	0.22341	0.22428	0.208583	0.345694	0.420301	-0.19886
40	1	0.345224	0.267593	0.345265	0.0905	-0.04858	0.354194	0.079069	0.186627	-0.019	0.399105
5	1	0.647073	0.269697	0.099627	0.015395	-0.03179	0.435287	-0.05749	0.18733	0.427895	0.006981
29	1	0.714511	0.238573	-0.05944	0.172083	-0.06573	0.761177	-0.06808	0.102641	0.180242	0.024021
39	1	0.57843	0.00016	0.256936	0.122583	0.041891	0.668413	0.025139	-0.07614	0.331133	0.051455
17	1	0.263631	-0.22461	0.336639	0.089977	0.534365	0.303924	0.37467	0.120411	0.104686	0.096309
7	1	0.667289	0.179909	0.227811	0.026981	-0.10199	0.508352	-0.08972	0.327374	0.208868	0.045126
21	1	0.263536	0.296558	0.17499	0.142245	0.122671	0.355958	-0.10643	-0.23032	0.106194	0.8746
11	1	0.723453	0.281044	0.048952	0.024766	-0.07822	0.550154	-0.07246	-0.08445	0.206589	0.400166
62	2	0.069806	0.796336	0.332443	-0.08412	-0.11447	-0.0309	1.03785	0.241953	-0.17347	-0.07544
59	2	0.147833	1.1561	-0.24867	0.057173	-0.11243	-0.01725	1.04545	0.019415	0.110953	-0.15856
56	2	-0.24548	0.917336	-0.26045	0.034118	0.554473	-0.04646	0.753504	0.018154	0.280089	-0.00529
72	2	0.013535	0.84695	0.057169	0.067689	0.014659	0.080162	1.04596	-0.34099	0.238562	-0.02369
44	2	-0.0405	0.6511	0.233068	0.117492	0.038844	0.137569	0.473091	0.202538	0.043	0.143801
45	2	0.341618	0.735057	0.07599	0.061759	-0.21442	0.128847	0.531111	0.187831	0.19593	-0.04372
63	2	-0.24319	1.26425	-0.22507	-0.14048	0.344485	-0.17847	0.628842	0.112098	0.126205	0.311323
41	2	0.192673	0.509964	-0.04895	0.118889	0.22743	0.317974	0.248314	0.050769	0.407933	-0.02499
51	2	0.596993	0.597665	0.113568	0.067675	-0.3759	0.649795	0.516496	0.068071	0.003744	-0.23811
79	2	0.092682	0.873614	-0.34486	0.164715	0.213852	0.09538	0.804192	-0.34785	0.268529	0.179751
48	2	0.17912	0.557753	0.250903	0.048879	-0.03665	0.257213	0.411336	-0.00043	0.257086	0.074794
76	2	0.258769	0.500501	0.055079	0.167478	0.018174	0.207711	0.794502	0.130637	-0.15627	0.023419
68	2	0.358773	0.38942	0.15305	0.134993	-0.03624	0.229315	0.356423	0.437791	0.111426	-0.13496
55	2	0.186728	0.604645	0.085441	0.074265	0.048921	0.242068	0.557674	0.209597	0.076641	-0.08598
61	2	-0.37281	0.772932	0.450286	-0.00906	0.158659	-0.11284	0.746934	0.21529	0.255519	-0.1049
74	2	0.329565	0.62186	0.229073	-0.05508	-0.12541	0.08664	0.940931	0.09133	-0.20405	0.085147
70	2	-0.24673	0.816656	0.30424	0.160327	-0.03449	-0.22834	0.68916	-0.065	0.444426	0.159757
54	2	0.28297	0.509754	0.217188	0.016925	-0.02684	0.204841	0.640622	0.164782	0.119491	-0.12974
66	2	-0.19453	1.26225	0.109375	-0.329	0.151908	-0.2715	1.04902	0.079207	-0.08073	0.223997

64	2	-0.09584	0.537856	0.499604	0.022184	0.0362	-0.2319	0.596526	0.090629	0.432636	0.112107
57	2	-0.07244	0.876781	-0.08137	0.156307	0.120715	-0.11396	0.76616	0.010082	0.21541	0.122306
75	2	0.039889	0.851868	0.012874	0.166448	-0.07108	0.184991	0.911434	0.21267	-0.17824	-0.13086
47	2	0.153202	0.566339	0.307304	0.007739	-0.03458	0.03282	0.472803	0.227146	0.150952	0.116278
77	2	0.201928	0.575064	-0.00123	0.25134	-0.0271	-0.10929	0.79778	0.227295	-0.16446	0.248666
53	2	0.072979	0.759376	0.1684	-0.01323	0.012479	0.003281	0.600335	0.289205	0.108394	-0.00121
50	2	-0.04229	0.690368	0.223264	-0.18263	0.311291	-0.02089	0.336585	0.138437	0.313807	0.232064
101	3	0.518498	-0.42517	0.488564	0.275332	0.142771	0.00779	0.155396	0.732153	0.054782	0.049879
91	3	0.311961	0.243343	0.416392	-0.28897	0.317271	0.287735	0.416394	0.335789	-0.24722	0.207303
90	3	-0.05535	-0.13039	0.47288	0.185251	0.527616	-0.19887	-0.05055	0.223964	0.442282	0.583168
87	3	0.49801	-0.07593	0.287804	0.015506	0.274609	0.210897	0.310405	0.251664	-0.02299	0.250023
99	3	0.148937	0.180723	0.679248	0.131564	-0.14047	-0.06287	0.426959	0.689509	0.180516	-0.23411
114	3	0.242932	0.136059	0.83624	-0.01202	-0.20321	0.371691	-0.12072	0.768564	0.000989	-0.02052
116	3	0.15505	0.444771	0.894134	0.086516	-0.58047	0.314057	-0.02112	0.819186	-0.1093	-0.00282
102	3	0.130378	0.046267	0.357503	0.317604	0.148249	-0.16631	0.147852	0.691012	0.06181	0.265637
104	3	0.296567	0.083874	0.690212	-0.12148	0.050826	-0.06158	-0.08246	1.18766	-0.19649	0.152871
83	3	0.443351	0.108669	0.516508	-0.22135	0.152823	0.149471	0.31844	0.539185	-0.06029	0.053191
110	3	0.088642	0.146678	0.617419	0.164083	-0.01682	0.144773	-0.08346	0.917945	0.167049	-0.14631
108	3	0.398477	0.14524	0.539718	-0.04363	-0.03981	0.181061	0.069546	0.869504	-0.07003	-0.05008
81	3	0.232887	0.134995	0.289219	0.009594	0.333305	-0.13159	0.393473	0.596458	-0.04671	0.188369
107	3	0.265582	-0.0708	0.655815	0.012989	0.136412	0.033563	0.055143	0.787277	0.153752	-0.02973
112	3	0.037427	0.173097	0.793042	0.164842	-0.16841	0.355083	-0.24481	0.885204	0.159616	-0.15509
113	3	0.035779	0.420523	1.08119	-0.21221	-0.32528	0.421198	0.055028	0.646507	-0.00478	-0.11795
97	3	-0.1794	-0.00048	0.665285	0.424461	0.090126	-0.36052	-0.14203	1.18333	0.688583	-0.36936
85	3	0.213486	-0.04318	0.530776	0.125265	0.173654	-0.02251	0.257258	0.580134	0.143384	0.041736
109	3	0.066657	0.362102	0.304432	-0.06219	0.329002	0.085945	0.004771	0.762973	0.026272	0.12004
105	3	-0.14302	0.025219	0.810688	0.03887	0.26824	-0.30469	-0.03893	1.07937	-0.06039	0.324637
100	3	0.021333	0.056647	0.485885	0.389877	0.046258	-0.2839	0.387341	0.79778	0.157468	-0.05869
111	3	-0.18207	0.413202	0.697861	0.002914	0.068096	0.033835	-0.05232	0.670692	0.164965	0.182824
96	3	0.338077	0.213843	0.415511	-0.09889	0.131457	0.195256	0.541771	0.671002	-0.26251	-0.14552
106	3	0.097341	0.0784	0.565158	0.1564	0.102701	0.17908	-0.03273	0.664721	-0.00356	0.192487
152	4	0.220966	0.098181	-0.04439	0.697809	0.027432	0.224681	0.238824	0.048087	0.389437	0.098971
121	4	0.08714	0.059315	-0.02632	0.950082	-0.07022	0.488248	0.197384	-0.44574	1.03254	-0.27243
125	4	-0.13997	0.199927	0.04262	1.16397	-0.26655	-0.06456	0.110432	0.094822	0.607269	0.252037
128	4	-0.12569	-0.09038	-0.11881	1.09464	0.240248	0.210784	0.155095	-0.06024	0.532153	0.16221
145	4	0.159007	-0.33164	0.517538	0.606769	0.048325	0.554847	-0.17713	0.320438	0.449367	-0.14753
156	4	0.266347	0.086221	-0.08778	0.443714	0.291498	0.103228	0.331342	-0.1165	0.588102	0.093824
126	4	-0.12785	0.12853	0.021018	1.11711	-0.13881	0.120524	0.116157	-0.0147	0.507858	0.270161
131	4	-0.00437	0.241123	-0.1627	0.822945	0.103001	0.333078	0.1578	-0.01491	0.548278	-0.02424
117	4	-0.21879	-0.0586	0.540951	0.800981	-0.06455	0.265847	0.228535	-0.06963	0.870496	-0.29525
122	4	-0.15685	-0.30607	0.321591	1.2125	-0.07117	0.122396	0.252071	-0.27698	1.00587	-0.10336
149	4	0.372967	-0.01622	-0.1589	0.567867	0.234286	0.388376	-0.02156	0.031784	0.331408	0.269988
153	4	0.191911	-0.11024	0.25449	0.47051	0.193329	0.271237	0.241421	0.178268	0.378666	-0.06959
146	4	0.045747	-0.12237	0.07719	0.734304	0.265131	0.303604	0.014576	0.102926	0.370598	0.208295
123	4	0.066703	0.087618	0.10213	0.935766	-0.19222	0.450782	0.09648	0.173474	0.391739	-0.11248
139	4	0.290648	-0.1555	0.132525	0.472654	0.259674	0.325629	0.095102	0.195467	0.491324	-0.10752
140	4	0.117028	0.112366	-0.02324	0.525901	0.267943	0.120667	0.105382	0.137568	0.489084	0.147299
137	4	0.248743	0.241418	-0.12531	0.402048	0.233099	0.134088	0.080834	0.256859	0.463582	0.064637
144	4	0.373611	0.017219	0.052188	0.546546	0.010437	0.313355	0.108261	0.234356	0.280045	0.063982

130	4	-0.09667	-0.05227	0.24525	0.989163	-0.08547	-0.03017	0.258285	0.22311	0.430382	0.11839
118	4	0.108706	0.060272	0.340164	0.779646	-0.28879	0.243957	0.264101	-0.056	0.821325	-0.27338
148	4	0.094375	0.31175	-0.31837	0.623393	0.288851	0.328862	-0.10675	-0.06688	0.603427	0.241342
134	4	-0.09712	0.296689	0.070689	0.288835	0.44091	0.06538	-0.05732	0.069886	0.741329	0.180729
141	4	0.088944	0.20967	-0.10376	0.700544	0.104603	0.16625	0.049714	0.287238	0.485926	0.010872
136	4	-0.01319	0.152442	0.332056	0.287453	0.241238	0.179078	0.135263	-0.09566	0.741211	0.040104
154	4	0.053577	0.131038	0.076115	0.742344	-0.00307	0.172235	0.198154	0.168072	0.299163	0.162376
129	4	0.012429	-0.10771	-0.51662	1.58926	0.022643	0.158029	0.131066	0.012082	0.571852	0.126971
192	5	0.045045	0.303987	-0.17946	0.205694	0.624737	0.179493	0.126263	0.040185	-0.13657	0.790627
171	5	0.153478	-0.21874	0.002961	0.136928	0.92537	0.059048	-0.13445	0.098703	0.226068	0.750636
169	5	0.15287	0.13835	0.178507	0.06857	0.461702	0.122156	0.182507	-0.08442	0.318285	0.461474
162	5	-0.06603	-0.05353	0.200841	0.080202	0.838513	0.046101	-0.24156	0.116314	0.314198	0.764942
182	5	0.297341	0.059631	-0.1574	0.087702	0.712724	0.664539	-0.03161	-0.10775	-0.06473	0.539545
190	5	-0.17064	-0.09406	0.21053	0.199505	0.854659	0.20375	0.41473	-0.08457	0.044038	0.42205
167	5	0.452084	-0.23449	-0.14273	0.104806	0.820329	0.316709	-0.23563	0.005872	0.17333	0.739717
178	5	0.335534	0.22599	0.02162	-0.21056	0.627419	-0.14606	0.347412	0.017567	-0.24102	1.0221
196	5	0.168042	0.158922	-0.14942	0.294051	0.528405	0.424239	0.029618	0.135316	-0.20361	0.614433
165	5	-0.11948	-0.15932	0.355431	0.159255	0.76411	0.227291	-0.24039	0.113361	0.141702	0.758036
187	5	0.194886	-0.14891	0.055436	0.189407	0.709182	0.112164	0.37043	-0.20634	0.029898	0.693847
191	5	0.430546	0.007665	-0.15082	0.091715	0.620891	0.362537	0.158543	-0.0398	-0.13907	0.657789
184	5	0.403158	0.011261	0.027884	-0.00828	0.565982	-0.06412	0.457224	-0.03099	-0.20142	0.839297
161	5	-0.01886	0.151263	0.051316	0.193404	0.622875	0.129296	-0.30984	0.311127	0.120232	0.749185
180	5	0.104637	0.027924	0.273902	-0.01173	0.605265	0.226382	0.093822	0.014425	-0.04916	0.714534
175	5	0.216203	-0.0196	0.148931	-0.00108	0.655538	-0.02863	-0.18238	0.323509	-0.07612	0.963621
179	5	0.304481	0.118747	0.109794	-0.08797	0.554944	-0.01717	0.223902	0.017837	-0.13599	0.911419
168	5	0.040938	-0.11786	0.329554	-0.00804	0.755402	0.171429	0.131693	0.013188	0.25941	0.42428
189	5	0.105481	0.050192	0.013342	0.135355	0.695631	0.389466	0.071753	0.170491	-0.07058	0.438871
173	5	-0.00473	0.117622	0.154575	-0.05023	0.782764	0.078415	-0.32747	0.088968	0.4053	0.754784
194	5	-0.09678	0.212325	-0.08364	0.147566	0.820529	-0.07875	0.035836	0.145433	-0.06855	0.966034
195	5	-0.28873	0.243065	-0.03799	0.258926	0.824728	-0.01556	0.128433	0.102436	-0.1065	0.891189
170	5	-0.06163	0.176812	0.192059	0.049122	0.643632	0.198648	0.06968	-0.10539	0.348958	0.488106
174	5	0.204262	0.109093	0.071775	0.02702	0.587851	0.004053	-0.14426	0.249046	-0.12368	1.01483
163	5	-0.08703	-0.02133	0.138442	0.036027	0.933892	0.155561	-0.13872	-0.07477	0.29717	0.760757
181	5	0.059967	-0.00256	0.183503	0.004424	0.754665	0.064694	0.020424	0.052214	0.044811	0.817858

Table S9

Sample number	Class label	FT-MIR-RFE tree votes (Validation set)					NIR-RFE tree votes (Validation set)				
		Class 1	Class 2	Class 3	Class 4	Class 5	Class 1	Class 2	Class 3	Class 4	Class 5
4	1	0.664506	0.128447	0.116987	0.021988	0.068072	0.216662	0.169155	0.427005	0.19068	-0.0035
6	1	0.313492	0.222769	0.260659	0.12851	0.07457	0.226077	0.046091	0.23346	0.398275	0.096097
8	1	0.38049	0.110032	0.259207	0.151489	0.098782	0.49334	0.07748	0.055817	0.117655	0.255709
10	1	0.69926	0.205132	0.061009	0.095527	-0.06093	0.398066	-0.09554	0.159842	0.043586	0.494047
13	1	0.409535	0.079527	0.428455	0.174025	-0.09154	0.241067	0.187019	0.301599	0.396407	-0.12609
15	1	0.341456	0.25769	0.282625	0.14334	-0.02511	-0.02017	0.26035	0.539185	0.116776	0.103859
22	1	0.329123	0.061803	0.095351	0.131135	0.382588	0.424796	0.011341	0.134473	0.320124	0.109266
24	1	0.1811	0.084924	0.113224	0.296577	0.324176	0.43463	0.139985	-0.26904	0.400407	0.294019
28	1	0.700552	-0.09657	0.26453	0.024952	0.106532	0.685596	-0.1154	0.170949	0.259584	-0.00073
30	1	0.587704	0.001952	0.126473	0.075234	0.208637	0.448287	-0.02943	0.034928	0.22122	0.324997
32	1	0.376152	-0.18712	0.443266	-0.14578	0.513477	0.560264	-0.08915	-0.10673	0.390538	0.24508
34	1	0.860823	0.002089	0.04134	0.206688	-0.11094	0.67851	-0.00761	0.056025	0.132346	0.140732
35	1	0.547662	0.007288	0.194805	0.028354	0.221891	0.696625	-0.16598	-0.2043	0.472505	0.201155
37	1	0.229768	0.190704	0.477647	-0.09488	0.196756	0.717618	-0.11118	0.031367	0.152679	0.209511
42	2	0.143257	0.557513	0.018446	0.206322	0.074463	0.299874	0.258711	0.232912	0.271184	-0.06268
43	2	0.115522	0.518498	0.05211	0.40476	-0.09089	0.097619	0.141575	0.328192	0.238312	0.194302
46	2	-0.07638	0.664893	0.090504	0.137323	0.183661	-0.05196	0.21422	0.188733	0.358966	0.290038
49	2	0.149025	0.638696	0.230456	-0.10246	0.084281	0.04591	0.482838	0.205045	0.008155	0.258052
52	2	-0.04189	0.730004	0.048387	0.06437	0.199128	-0.10027	0.470362	0.511243	-0.01952	0.138186
58	2	0.023954	0.685654	0.024424	0.236965	0.029003	-0.00374	0.493596	0.205947	0.388628	-0.08443
60	2	-0.09184	0.813608	0.128369	0.146934	0.002934	-0.01276	0.894189	0.152878	0.012627	-0.04693
65	2	-0.09705	1.05708	0.426178	-0.38788	0.001677	-0.09907	0.843398	-0.03692	0.304625	-0.01203
67	2	0.02309	0.507159	0.27836	0.198099	-0.00671	-0.02654	0.731479	0.079219	-0.01897	0.234816
69	2	-0.0263	0.440001	0.369211	0.172697	0.044391	-0.0478	0.726968	0.271192	-0.01598	0.065617
71	2	-0.11477	1.08097	-0.0274	-0.25024	0.311435	0.168503	1.01717	-0.20949	0.069606	-0.04579
73	2	0.058252	0.56228	0.057355	0.331093	-0.00898	0.060769	0.641365	0.084975	0.300247	-0.08736
78	2	-0.0467	0.718029	0.112186	0.127177	0.089304	0.049207	0.717196	0.138627	0.06793	0.02704
80	2	0.081995	0.482237	0.055704	0.377424	0.002639	-0.17077	0.627173	0.205489	-0.06165	0.399762
82	3	0.291247	0.011712	0.429501	-0.09608	0.363618	-0.07737	0.304962	0.622671	0.078392	0.071344
84	3	0.17603	0.117114	0.335909	-0.0111	0.38205	0.004162	0.290789	0.521412	-0.08887	0.272505
86	3	0.357298	-0.05683	0.423914	0.034543	0.241077	-0.01557	0.323582	0.358749	-0.0688	0.402037
88	3	-0.07436	0.101788	0.308392	0.127021	0.537155	-0.08859	0.219957	0.238422	0.288735	0.341474
89	3	-0.07459	0.111942	0.429719	0.018421	0.514504	0.066803	0.029017	0.155554	0.381704	0.366922
92	3	0.363876	0.075258	0.174348	0.214456	0.172061	0.298574	0.362225	-0.01545	-0.03654	0.391191
93	3	0.313185	0.190407	0.343635	-0.01838	0.171156	0.340245	0.22358	0.334547	-0.10178	0.203406
94	3	0.128483	0.016398	0.38236	-0.00084	0.473602	0.209066	-0.12246	0.477604	-0.00464	0.44043
95	3	0.274732	0.162092	0.277976	0.042999	0.242201	0.386207	0.170713	0.227607	-0.15948	0.374956
98	3	-0.05312	0.374661	0.351852	0.507653	-0.18105	-0.20491	0.300432	0.662512	0.274319	-0.03235
103	3	-0.07789	-0.17806	0.86465	0.227626	0.163677	0.030207	-0.06309	0.6594	0.484534	-0.11105
115	3	0.072103	0.351787	0.651214	0.152835	-0.22794	0.183187	0.101171	0.496455	-0.04663	0.265821
119	4	-0.16855	0.306349	0.571546	0.880662	-0.59001	0.235746	0.18439	-0.0167	0.696476	-0.09992
120	4	-0.1966	0.33391	0.430473	0.687388	-0.25517	0.258702	0.023566	-0.10318	0.937721	-0.11681
124	4	0.113878	0.052264	-0.09245	1.08959	-0.16328	0.397107	-0.08235	-0.15264	0.785178	0.052702
127	4	-0.162	0.403021	0.334774	0.874709	-0.45051	0.146038	0.117198	0.066928	0.511512	0.158324
132	4	-0.01358	-0.11604	-0.16514	1.29782	-0.00306	0.261578	0.185622	-0.02312	0.61027	-0.03435
133	4	-0.10104	0.126	-0.00468	1.35143	-0.37172	0.206509	0.074889	0.202944	0.616301	-0.10064

135	4	0.065455	0.135884	0.148545	0.24372	0.406396	0.244207	-0.0045	0.13046	0.571515	0.058318
138	4	-0.10896	-0.08749	0.076452	0.584002	0.535988	0.028701	0.13061	0.210994	0.502903	0.126792
142	4	0.217487	-0.02954	-0.03306	0.73536	0.109757	0.094348	0.145633	0.339305	0.338728	0.081986
143	4	0.036319	0.131616	-0.01135	0.795497	0.047923	0.130718	0.184084	-0.0075	0.49035	0.202344
147	4	0.090075	0.111291	-0.07531	0.667232	0.206716	0.413356	-0.04135	0.079797	0.491764	0.056434
150	4	0.23395	-0.09598	-0.05525	0.749494	0.167781	0.317346	0.098264	-0.14276	0.529732	0.197418
151	4	0.122787	-0.15813	0.258684	0.490809	0.285847	0.371648	-0.10693	-0.07376	0.396166	0.412874
155	4	0.108316	-0.00215	0.047942	0.675427	0.170462	0.408456	0.147782	-0.09558	0.548258	-0.00891
157	5	-0.10275	0.186372	0.111331	0.255158	0.549886	-0.01501	-0.19163	0.329509	0.402718	0.474411
158	5	0.003476	0.047506	0.181729	-0.0134	0.780686	0.014924	-0.09422	0.074015	0.375662	0.629621
159	5	-0.12933	-0.08012	0.182163	0.23118	0.796111	0.032458	-0.08228	0.245162	0.265881	0.538776
160	5	-0.02432	0.239534	-0.01757	-0.07819	0.880541	0.063869	-0.08531	0.121458	0.278859	0.621126
164	5	-0.01968	0.143177	0.113072	0.025733	0.737694	0.260038	-0.24368	0.013723	0.213349	0.756574
166	5	0.205136	-0.3314	0.069099	0.125318	0.931843	0.252922	-0.19167	-0.21591	0.527078	0.627581
172	5	-0.10792	0.206324	0.243232	0.031233	0.627134	0.174585	-0.10635	-0.06271	0.290623	0.703858
176	5	0.206899	-0.01467	0.275393	0.051155	0.481223	0.270145	-0.29231	0.007889	0.104389	0.909883
177	5	0.249439	0.124314	0.213315	-0.18131	0.594244	-0.04355	0.187204	0.419938	-0.39878	0.835188
183	5	0.254654	-0.20523	0.318605	-0.11214	0.744117	0.069709	0.36673	0.082023	-0.13923	0.620767
185	5	0.002781	-0.10391	0.155758	0.162124	0.783244	-0.00998	0.160645	0.224436	-0.06377	0.688673
186	5	0.05686	0.067207	0.093356	0.017576	0.765001	0.147734	0.153502	0.129139	0.011108	0.558517
188	5	0.04757	-0.12341	0.128612	0.278915	0.66831	-0.05361	0.154171	0.101309	-0.02299	0.82112
193	5	-0.06899	0.102048	-0.27359	0.360997	0.879535	-0.05629	0.15743	0.002371	0.002914	0.893578

Table S10

Sample number	Class label	FT-MIR-Bo tree votes (Calibration set)					NIR-Bo tree votes (Calibration set)				
		Class 1	Class 2	Class 3	Class 4	Class 5	Class 1	Class 2	Class 3	Class 4	Class 5
36	1	0.271739	0.121739	0.15	0.147826	0.308696	0.394286	0.077143	0.085714	0.151429	0.291429
33	1	0.783333	0.05	0.090476	0.028571	0.047619	0.469453	0.096463	0.057878	0.038585	0.337621
12	1	0.339853	0.180929	0.283619	0.0978	0.0978	0.354098	0.111475	0.15082	0.15082	0.232787
1	1	0.482843	0.068627	0.196078	0.208333	0.044118	0.30303	0.024242	0.060606	0.075758	0.536364
2	1	0.231308	0.149533	0.296729	0.247664	0.074766	0.421725	0.009585	0.044728	0.083067	0.440895
20	1	0.603248	0.055684	0.074246	0.046404	0.220418	0.23125	0.284375	0.165625	0.225	0.09375
19	1	0.606299	0.07874	0.178478	0.060367	0.076115	0.216049	0.395062	0.209877	0.154321	0.024691
27	1	0.942661	0.020642	0.016055	0.006881	0.013761	0.303681	0.147239	0.079755	0.070552	0.398773
16	1	0.406181	0.050773	0.064018	0.099338	0.379691	0.21519	0.151899	0.189873	0.420886	0.022152
31	1	0.308756	0.290323	0.096774	0.115207	0.18894	0.432927	0.054878	0.073171	0.060976	0.378049
3	1	0.579572	0.090261	0.182898	0.078385	0.068884	0.44373	0.138264	0.083601	0.057878	0.276527
18	1	0.788698	0.056511	0.029484	0.031941	0.093366	0.24058	0.327536	0.249275	0.13913	0.043478
9	1	0.783333	0.069048	0.035714	0.05	0.061905	0.557377	0.055738	0.140984	0.039344	0.206557
26	1	0.430288	0.257212	0.127404	0.052885	0.132212	0.551205	0.126506	0.135542	0.036145	0.150602
25	1	0.258993	0.136691	0.141487	0.172662	0.290168	0.462264	0.059748	0.147799	0.122642	0.207547
23	1	0.29397	0.170854	0.10804	0.288945	0.138191	0.231844	0.173184	0.187151	0.217877	0.189944
38	1	0.492857	0.154762	0.097619	0.121429	0.133333	0.550633	0.088608	0.117089	0.060127	0.183544
14	1	0.586797	0.02934	0.09291	0.117359	0.173594	0.175953	0.266862	0.199413	0.334311	0.02346
40	1	0.288136	0.2954	0.16707	0.121065	0.128329	0.444795	0.044164	0.116719	0.041009	0.353312
5	1	0.904328	0.036446	0.038724	0.002278	0.018223	0.273927	0.174917	0.125413	0.366337	0.059406
29	1	0.785888	0.072993	0.058394	0.036496	0.046229	0.686335	0.071429	0.059006	0.031056	0.152174
39	1	0.746445	0.042654	0.059242	0.054502	0.097156	0.52	0.153846	0.113846	0.061538	0.150769
17	1	0.422111	0.050251	0.082915	0.123116	0.321608	0.250765	0.351682	0.201835	0.12844	0.067278
7	1	0.679707	0.09291	0.09291	0.046455	0.08802	0.454259	0.164038	0.160883	0.135647	0.085174
21	1	0.193925	0.13785	0.193925	0.280374	0.193925	0.164087	0.027864	0.052632	0.099071	0.656347
11	1	0.835381	0.063882	0.051597	0.029484	0.019656	0.51227	0.021472	0.116564	0.110429	0.239264
62	2	0.050125	0.493734	0.298246	0.105263	0.052632	0.056782	0.766562	0.141956	0.031546	0.003155
59	2	0.22381	0.338095	0.169048	0.092857	0.17619	0.123418	0.655063	0.126582	0.06962	0.025316
56	2	0.137116	0.392435	0.153664	0.092199	0.224586	0.177177	0.576577	0.135135	0.084084	0.027027
72	2	0.042289	0.58209	0.171642	0.077114	0.126866	0.220126	0.518868	0.081761	0.122642	0.056604
44	2	0.044706	0.416471	0.322353	0.112941	0.103529	0.151976	0.534954	0.148936	0.066869	0.097264
45	2	0.209412	0.322353	0.247059	0.105882	0.115294	0.159722	0.482639	0.236111	0.100694	0.020833
63	2	0.044665	0.424318	0.208437	0.230769	0.091811	0.137931	0.545455	0.188088	0.087774	0.040752
41	2	0.1575	0.0675	0.1625	0.245	0.3675	0.186495	0.122186	0.180064	0.469453	0.041801
51	2	0.362319	0.379227	0.193237	0.021739	0.043478	0.363636	0.38558	0.134796	0.025078	0.090909
79	2	0.07767	0.43932	0.106796	0.058252	0.317961	0.145631	0.763754	0.02589	0.048544	0.016181
48	2	0.105012	0.546539	0.178998	0.052506	0.116945	0.204142	0.535503	0.109467	0.115385	0.035503
76	2	0.096852	0.610169	0.106538	0.050847	0.135593	0.169811	0.606918	0.119497	0.056604	0.04717
68	2	0.192771	0.20241	0.274699	0.260241	0.06988	0.191824	0.41195	0.320755	0.053459	0.022013
55	2	0.235431	0.414918	0.102564	0.090909	0.156177	0.152027	0.516892	0.199324	0.094595	0.037162
61	2	0.020179	0.428251	0.219731	0.278027	0.053812	0.104762	0.374603	0.273016	0.209524	0.038095
74	2	0.272727	0.433014	0.143541	0.033493	0.117225	0.110738	0.684564	0.161074	0.016779	0.026846
70	2	0.00885	0.471239	0.20354	0.245575	0.070796	0.110063	0.374214	0.103774	0.374214	0.037736
54	2	0.278195	0.338346	0.20802	0.100251	0.075188	0.234043	0.531915	0.12462	0.045593	0.06383
66	2	0.01432	0.761337	0.109785	0.042959	0.071599	0.072131	0.57377	0.22623	0.085246	0.042623
64	2	0.025761	0.555035	0.217799	0.145199	0.056206	0.155556	0.219048	0.136508	0.450794	0.038095

57	2	0.035	0.6825	0.095	0.065	0.1225	0.084746	0.59887	0.138418	0.161017	0.016949
75	2	0.070423	0.607981	0.096244	0.084507	0.140845	0.122449	0.664723	0.166181	0.011662	0.034985
47	2	0.115207	0.4447	0.290323	0.073733	0.076037	0.214953	0.489097	0.146417	0.102804	0.046729
77	2	0.081146	0.4821	0.116945	0.088305	0.231504	0.148607	0.452012	0.216718	0.117647	0.065015
53	2	0.151927	0.519274	0.124717	0.063492	0.14059	0.20059	0.374631	0.253687	0.112094	0.058997
50	2	0.027972	0.662005	0.177156	0.027972	0.104895	0.148515	0.237624	0.115512	0.389439	0.108911
101	3	0.195	0.1075	0.205	0.305	0.1875	0.06135	0.165644	0.546012	0.190184	0.03681
91	3	0.17713	0.201794	0.443946	0.056054	0.121076	0.33125	0.271875	0.140625	0.125	0.13125
90	3	0.028571	0.202381	0.302381	0.292857	0.17381	0.085627	0.055046	0.088685	0.504587	0.266055
87	3	0.286047	0.127907	0.427907	0.055814	0.102326	0.336634	0.231023	0.194719	0.09901	0.138614
99	3	0.135321	0.33945	0.376147	0.09633	0.052752	0.192429	0.451104	0.236593	0.113565	0.006309
114	3	0.064356	0.405941	0.35396	0.056931	0.118812	0.174917	0.072607	0.541254	0.089109	0.122112
116	3	0.081281	0.211823	0.455665	0.20936	0.041872	0.11465	0.079618	0.643312	0.063694	0.098726
102	3	0.066059	0.093394	0.296128	0.357631	0.186788	0.080997	0.158879	0.4081	0.320872	0.031153
104	3	0.273632	0.161692	0.380597	0.109453	0.074627	0.080386	0.041801	0.749196	0.099678	0.028939
83	3	0.266504	0.070905	0.484108	0.07824	0.100244	0.27	0.37	0.283333	0.06	0.016667
110	3	0.07732	0.237113	0.311856	0.185567	0.188144	0.053892	0.062874	0.640719	0.221557	0.020958
108	3	0.15	0.197619	0.188095	0.147619	0.316667	0.104101	0.07571	0.637224	0.107256	0.07571
81	3	0.066327	0.094388	0.543367	0.147959	0.147959	0.168605	0.293605	0.360465	0.113372	0.063953
107	3	0.12529	0.194896	0.366589	0.222738	0.090487	0.104895	0.125874	0.51049	0.206294	0.052448
112	3	0.030151	0.160804	0.51005	0.251256	0.047739	0.107843	0.01634	0.578431	0.169935	0.127451
113	3	0.067757	0.278037	0.567757	0.051402	0.035047	0.29878	0.094512	0.439024	0.07622	0.091463
97	3	0.018648	0.097902	0.317016	0.475524	0.090909	0.124242	0.093939	0.19697	0.533333	0.051515
85	3	0.03423	0.180929	0.550122	0.151589	0.08313	0.28882	0.31677	0.204969	0.161491	0.02795
109	3	0.19426	0.19426	0.181015	0.18543	0.245033	0.140299	0.056716	0.573134	0.137313	0.092537
105	3	0.00232	0.099768	0.470998	0.238979	0.187935	0.041812	0.052265	0.574913	0.264808	0.066202
100	3	0.053528	0.107056	0.391727	0.347932	0.099757	0.12963	0.194444	0.25	0.404321	0.021605
111	3	0.043902	0.131707	0.497561	0.243902	0.082927	0.060606	0.036364	0.5	0.354545	0.048485
96	3	0.147619	0.416667	0.328571	0.028571	0.078571	0.175926	0.509259	0.228395	0.064815	0.021605
106	3	0.139706	0.159314	0.404412	0.191176	0.105392	0.305994	0.091483	0.208202	0.11041	0.283912
152	4	0.147059	0.198529	0.394608	0.147059	0.112745	0.219436	0.297806	0.159875	0.294671	0.028213
121	4	0.132678	0.036855	0.100737	0.673219	0.056511	0.080745	0.142857	0.040373	0.708075	0.02795
125	4	0.009324	0.107226	0.100233	0.7669	0.016317	0.02994	0.05988	0.191617	0.640719	0.077844
128	4	0.016355	0.067757	0.119159	0.757009	0.03972	0.085106	0.051672	0.18541	0.607903	0.069909
145	4	0.221662	0.073048	0.279597	0.34005	0.085642	0.313333	0.166667	0.213333	0.153333	0.153333
156	4	0.095122	0.112195	0.282927	0.373171	0.136585	0.194969	0.210692	0.141509	0.380503	0.072327
126	4	0.015982	0.114155	0.118721	0.726027	0.025114	0.210169	0.237288	0.084746	0.325424	0.142373
131	4	0.037879	0.146465	0.133838	0.636364	0.045455	0.267516	0.289809	0.066879	0.318471	0.057325
117	4	0.012407	0.042184	0.091811	0.826303	0.027295	0.042553	0.097264	0.133739	0.714286	0.012158
122	4	0.029197	0.097324	0.097324	0.712895	0.06326	0.081169	0.136364	0.038961	0.701299	0.042208
149	4	0.121951	0.090909	0.124169	0.27051	0.392461	0.180685	0.062305	0.218069	0.286604	0.252336
153	4	0.204651	0.095349	0.248837	0.316279	0.134884	0.159509	0.153374	0.303681	0.312883	0.070552
146	4	0.062972	0.025189	0.105793	0.609572	0.196474	0.272727	0.059441	0.132867	0.388112	0.146853
123	4	0.056511	0.078624	0.125307	0.707617	0.031941	0.276316	0.220395	0.213816	0.171053	0.118421
139	4	0.220183	0.06422	0.222477	0.341743	0.151376	0.143713	0.140719	0.224551	0.461078	0.02994
140	4	0.092417	0.151659	0.104265	0.265403	0.386256	0.036066	0.055738	0.190164	0.695082	0.022951
137	4	0.139089	0.088729	0.227818	0.28777	0.256595	0.10241	0.072289	0.225904	0.542169	0.057229
144	4	0.469907	0.099537	0.134259	0.108796	0.1875	0.223565	0.145015	0.287009	0.287009	0.057402
130	4	0.00489	0.02934	0.08313	0.872861	0.00978	0.070513	0.073718	0.163462	0.647436	0.044872

118	4	0.021378	0.092637	0.102138	0.755344	0.028504	0.123711	0.147766	0.161512	0.542955	0.024055
148	4	0.039702	0.064516	0.1067	0.60794	0.181141	0.096667	0.033333	0.116667	0.536667	0.216667
134	4	0.037037	0.164352	0.106481	0.19213	0.5	0.074627	0.047761	0.131343	0.653731	0.092537
141	4	0.034398	0.095823	0.201474	0.4914	0.176904	0.098101	0.088608	0.287975	0.462025	0.063291
136	4	0.060526	0.136842	0.242105	0.326316	0.234211	0.065672	0.026866	0.155224	0.707463	0.044776
154	4	0.034247	0.107306	0.296804	0.525114	0.03653	0.171429	0.22	0.22	0.322857	0.065714
129	4	0.018018	0.022523	0.047297	0.896396	0.015766	0.12844	0.04893	0.094801	0.562691	0.165138
192	5	0.086449	0.252336	0.151869	0.224299	0.285047	0.238411	0.059603	0.049669	0.023179	0.629139
171	5	0.064073	0.048055	0.139588	0.267735	0.480549	0.087662	0.016234	0.068182	0.11039	0.717532
169	5	0.189394	0.118687	0.219697	0.156566	0.315657	0.2125	0.109375	0.109375	0.24375	0.325
162	5	0.026379	0.035971	0.086331	0.136691	0.714628	0.049231	0.021538	0.129231	0.16	0.64
182	5	0.24	0.1825	0.1025	0.0675	0.4075	0.657224	0.033994	0.045326	0.022663	0.240793
190	5	0.091566	0.277108	0.113253	0.115663	0.40241	0.268786	0.193642	0.231214	0.078035	0.228324
167	5	0.379653	0.062035	0.064516	0.101737	0.39206	0.265625	0.021875	0.040625	0.04375	0.628125
178	5	0.181818	0.137592	0.228501	0.12285	0.329238	0.143312	0.047771	0.070064	0.035032	0.703822
196	5	0.232446	0.159806	0.135593	0.113801	0.358354	0.42492	0.022364	0.060703	0.019169	0.472843
165	5	0.059102	0.037825	0.120567	0.172577	0.609929	0.201923	0.038462	0.070513	0.147436	0.541667
187	5	0.088517	0.181818	0.105263	0.117225	0.507177	0.319876	0.055901	0.118012	0.136646	0.369565
191	5	0.407143	0.119048	0.152381	0.15	0.171429	0.40407	0.063953	0.069767	0.023256	0.438953
184	5	0.381546	0.134663	0.062344	0.029925	0.391521	0.245509	0.10479	0.131737	0.062874	0.45509
161	5	0.050602	0.06506	0.127711	0.277108	0.479518	0.117834	0.019108	0.079618	0.149682	0.633758
180	5	0.186224	0.30102	0.19898	0.053571	0.260204	0.360248	0.046584	0.068323	0.037267	0.487578
175	5	0.097902	0.072261	0.090909	0.132867	0.606061	0.096386	0.009036	0.090361	0.045181	0.759036
179	5	0.2525	0.0825	0.1375	0.1125	0.415	0.168675	0.036145	0.120482	0.069277	0.605422
168	5	0.048193	0.079518	0.151807	0.13012	0.590361	0.242331	0.131902	0.095092	0.214724	0.315951
189	5	0.167095	0.174807	0.041131	0.061697	0.55527	0.532308	0.049231	0.055385	0.030769	0.332308
173	5	0.045662	0.041096	0.093607	0.06621	0.753425	0.113889	0.025	0.088889	0.213889	0.558333
194	5	0.034169	0.300683	0.111617	0.157175	0.396355	0.117089	0.028481	0.079114	0.06962	0.705696
195	5	0.031325	0.301205	0.077108	0.13494	0.455422	0.205696	0.03481	0.03481	0.06962	0.655063
170	5	0.062356	0.124711	0.096998	0.2194	0.496536	0.102564	0.028846	0.147436	0.304487	0.416667
174	5	0.086957	0.052632	0.08238	0.15103	0.627002	0.093939	0.018182	0.039394	0.042424	0.806061
163	5	0.032558	0.046512	0.093023	0.139535	0.688372	0.148148	0.010101	0.043771	0.127946	0.670034
181	5	0.083135	0.185273	0.159145	0.071259	0.501188	0.211656	0.03681	0.067485	0.116564	0.567485

Table S11

Sample number	Class label	FT-MIR-Bo tree votes (Validation set)					NIR-Bo tree votes (Validation set)				
		Class 1	Class 2	Class 3	Class 4	Class 5	Class 1	Class 2	Class 3	Class 4	Class 5
4	1	0.584427	0.108486	0.208224	0.069991	0.028871	0.204571	0.264	0.344	0.166857	0.020571
6	1	0.348206	0.211724	0.172353	0.15748	0.110236	0.162286	0.105143	0.170286	0.505143	0.057143
8	1	0.44357	0.134733	0.134733	0.115486	0.171479	0.624	0.059429	0.091429	0.051429	0.173714
10	1	0.877515	0.051619	0.023622	0.016623	0.030621	0.458286	0.014857	0.085714	0.032	0.409143
13	1	0.369204	0.106737	0.158355	0.181977	0.183727	0.185143	0.243429	0.16	0.372571	0.038857
15	1	0.127734	0.122485	0.48119	0.167104	0.101487	0.168	0.178286	0.333714	0.24	0.08
22	1	0.325459	0.132108	0.077865	0.107612	0.356955	0.237714	0.131429	0.204571	0.284571	0.141714
24	1	0.40245	0.127734	0.090114	0.142607	0.237095	0.385143	0.051429	0.068571	0.28	0.214857
28	1	0.594926	0.154856	0.079615	0.067367	0.103237	0.652571	0.051429	0.104	0.068571	0.123429
30	1	0.728784	0.045494	0.051619	0.074366	0.099738	0.467429	0.046857	0.068571	0.060571	0.356571
32	1	0.258093	0.088364	0.199475	0.055118	0.39895	0.412571	0.048	0.043429	0.096	0.4
34	1	0.501312	0.131234	0.11636	0.125984	0.125109	0.501714	0.034286	0.179429	0.038857	0.245714
35	1	0.425197	0.074366	0.167104	0.125109	0.208224	0.580571	0.016	0.035429	0.090286	0.277714
37	1	0.568679	0.091864	0.079615	0.073491	0.186352	0.64	0.034286	0.042286	0.028571	0.254857
42	2	0.111986	0.251969	0.257218	0.223972	0.154856	0.212571	0.296	0.32	0.124571	0.046857
43	2	0.067367	0.296588	0.268591	0.252843	0.114611	0.221714	0.245714	0.204571	0.272	0.056
46	2	0.022747	0.585302	0.183727	0.114611	0.093613	0.117714	0.218286	0.118857	0.468571	0.076571
49	2	0.068241	0.748906	0.095363	0.027997	0.059493	0.122286	0.616	0.124571	0.068571	0.068571
52	2	0.025372	0.738408	0.083115	0.062117	0.090989	0.170286	0.340571	0.317714	0.102857	0.068571
58	2	0.088364	0.56343	0.093613	0.12336	0.131234	0.116571	0.361143	0.187429	0.308571	0.026286
60	2	0.03762	0.749781	0.092738	0.038495	0.081365	0.021714	0.891429	0.067429	0.011429	0.008
65	2	0.013998	0.76203	0.117235	0.049869	0.056868	0.114286	0.629714	0.054857	0.181714	0.019429
67	2	0.015748	0.75678	0.12161	0.079615	0.026247	0.123429	0.514286	0.219429	0.064	0.078857
69	2	0.023622	0.666667	0.160105	0.097113	0.052493	0.145143	0.580571	0.210286	0.043429	0.020571
71	2	0.059493	0.694663	0.103237	0.027122	0.115486	0.131429	0.725714	0.048	0.058286	0.036571
73	2	0.044619	0.596675	0.146107	0.107612	0.104987	0.181714	0.563429	0.096	0.145143	0.013714
78	2	0.034121	0.696413	0.11811	0.048994	0.102362	0.075429	0.763429	0.122286	0.030857	0.008
80	2	0.030621	0.463692	0.223097	0.156605	0.125984	0.169143	0.539429	0.138286	0.053714	0.099429
82	3	0.092738	0.066492	0.706037	0.057743	0.07699	0.173714	0.368	0.300571	0.123429	0.034286
84	3	0.055993	0.152231	0.629046	0.102362	0.060367	0.273143	0.2	0.294857	0.157714	0.074286
86	3	0.093613	0.067367	0.631671	0.127734	0.079615	0.290286	0.181714	0.299429	0.114286	0.114286
88	3	0.024497	0.188976	0.384952	0.218723	0.182852	0.074286	0.136	0.274286	0.465143	0.050286
89	3	0.026247	0.195101	0.458443	0.161855	0.158355	0.083429	0.067429	0.260571	0.485714	0.102857
92	3	0.090989	0.184602	0.584427	0.081365	0.058618	0.451429	0.131429	0.128	0.032	0.257143
93	3	0.086614	0.153106	0.629921	0.068241	0.062117	0.478857	0.112	0.169143	0.052571	0.187429
94	3	0.048119	0.073491	0.684164	0.106737	0.087489	0.361143	0.045714	0.214857	0.108571	0.269714
95	3	0.230096	0.108486	0.511811	0.093613	0.055993	0.486857	0.094857	0.137143	0.034286	0.246857
98	3	0.024497	0.124234	0.433946	0.367454	0.049869	0.124571	0.116571	0.315429	0.419429	0.024
103	3	0.036745	0.074366	0.468066	0.244969	0.175853	0.067429	0.056	0.373714	0.467429	0.035429
115	3	0.060367	0.179353	0.595801	0.11811	0.046369	0.130286	0.088	0.52	0.100571	0.161143
119	4	0.004374	0.165354	0.090114	0.724409	0.015748	0.064	0.072	0.117714	0.725714	0.020571
120	4	0.004374	0.052493	0.056868	0.87664	0.009624	0.062857	0.036571	0.08	0.797714	0.022857
124	4	0.010499	0.045494	0.065617	0.870516	0.007874	0.125714	0.088	0.075429	0.651429	0.059429
127	4	0.004374	0.07874	0.076115	0.832896	0.007874	0.060571	0.086857	0.146286	0.648	0.058286
132	4	0.014873	0.033246	0.04287	0.889764	0.019248	0.134857	0.145143	0.104	0.581714	0.034286
133	4	0.002625	0.019248	0.036745	0.937008	0.004374	0.030857	0.078857	0.131429	0.749714	0.009143

135	4	0.046369	0.079615	0.136483	0.372703	0.364829	0.132571	0.118857	0.075429	0.617143	0.056
138	4	0.027122	0.069991	0.092738	0.581802	0.228346	0.042286	0.051429	0.109714	0.754286	0.042286
142	4	0.075241	0.101487	0.090114	0.486439	0.246719	0.083429	0.090286	0.272	0.505143	0.049143
143	4	0.043745	0.07874	0.090114	0.663167	0.124234	0.094857	0.089143	0.074286	0.702857	0.038857
147	4	0.03937	0.03937	0.07699	0.771654	0.072616	0.101714	0.060571	0.249143	0.510857	0.077714
150	4	0.054243	0.055993	0.134733	0.656168	0.098863	0.117714	0.056	0.164571	0.521143	0.140571
151	4	0.036745	0.048994	0.307087	0.468066	0.139108	0.188571	0.029714	0.082286	0.321143	0.378286
155	4	0.020122	0.055118	0.252843	0.629921	0.041995	0.237714	0.196571	0.084571	0.434286	0.046857
157	5	0.027122	0.110236	0.164479	0.206474	0.491689	0.053714	0.026286	0.284571	0.328	0.307429
158	5	0.026247	0.027122	0.077865	0.106737	0.76203	0.067429	0.030857	0.124571	0.305143	0.472
159	5	0.020997	0.026247	0.051619	0.167104	0.734033	0.078857	0.027429	0.171429	0.332571	0.389714
160	5	0.031496	0.038495	0.053368	0.140857	0.735783	0.085714	0.014857	0.077714	0.241143	0.580571
164	5	0.033246	0.040245	0.096238	0.154856	0.675416	0.208	0.014857	0.038857	0.131429	0.606857
166	5	0.049869	0.034121	0.038495	0.058618	0.818898	0.062857	0.008	0.038857	0.147429	0.742857
172	5	0.034996	0.08399	0.099738	0.197725	0.583552	0.041143	0.011429	0.074286	0.146286	0.726857
176	5	0.090114	0.071741	0.100612	0.150481	0.587052	0.094857	0.009143	0.022857	0.025143	0.848
177	5	0.130359	0.132108	0.401575	0.068241	0.267717	0.273143	0.026286	0.139429	0.051429	0.509714
183	5	0.183727	0.029746	0.074366	0.057743	0.654418	0.285714	0.077714	0.110857	0.050286	0.475429
185	5	0.049869	0.156605	0.15748	0.107612	0.528434	0.198857	0.026286	0.064	0.044571	0.666286
186	5	0.165354	0.151356	0.173228	0.11811	0.391951	0.313143	0.059429	0.051429	0.038857	0.537143
188	5	0.036745	0.038495	0.106737	0.247594	0.570429	0.109714	0.028571	0.068571	0.092571	0.700571
193	5	0.013123	0.182852	0.062992	0.090989	0.650044	0.070857	0.011429	0.035429	0.046857	0.835429

Table S12

Sample number	Class label	FT-MIR-Bo tree votes (Calibration set)					NIR-Bo tree votes (Calibration set)				
		Class 1	Class 2	Class 3	Class 4	Class 5	Class 1	Class 2	Class 3	Class 4	Class 5
36	1	0.654234	-0.01061	0.139123	-0.13156	0.348814	0.558262	-0.06611	-0.15206	0.509428	0.150477
33	1	1.08857	0.135463	0.014791	-0.29841	0.059592	0.683707	0.089601	-0.26057	0.149138	0.338124
12	1	0.471664	0.199388	0.221603	0.103613	0.003732	0.611817	0.016568	0.007697	0.436495	-0.07258
1	1	0.886867	0.06461	0.046081	0.107999	-0.10556	0.501489	-0.15677	0.05261	0.205112	0.397561
2	1	0.543511	0.301225	0.037015	-0.011	0.129251	0.560756	-0.17693	-0.10106	0.321317	0.395913
20	1	0.728328	-0.25977	0.144513	-0.15779	0.544727	0.184416	0.40578	0.219011	0.363706	-0.17291
19	1	1.14971	-0.21834	0.137963	-0.01713	-0.0522	0.183581	0.41973	0.170877	0.31137	-0.08556
27	1	1.14539	0.073306	-0.16658	-0.19627	0.14415	0.625997	0.045978	0.036444	0.004207	0.287374
16	1	0.329149	-0.22428	0.25874	-0.11764	0.754038	0.095129	0.261465	0.280314	0.420817	-0.05772
31	1	0.686163	0.145887	-0.15045	0.124996	0.193405	0.583778	0.055969	-0.28243	0.384422	0.258263
3	1	0.739251	0.035354	0.086212	0.003229	0.135955	0.610129	0.150695	-0.0512	0.046899	0.243477
18	1	0.922362	-0.04008	0.11954	-0.10773	0.105915	0.408399	0.140683	0.389661	0.248582	-0.18732
9	1	0.56935	0.159188	0.171687	0.084871	0.014904	0.56064	-0.09128	0.295404	-0.01964	0.25487
26	1	0.915651	0.194781	0.081135	-0.09215	-0.09942	0.561807	0.175946	0.055112	0.08537	0.121766
25	1	0.480226	0.111549	-0.09195	0.122915	0.377264	0.642105	-0.125	0.016971	0.370187	0.095736
23	1	0.663607	0.246799	-0.20537	0.178378	0.116585	0.387196	0.237076	-0.14599	0.243321	0.278392
38	1	0.725266	0.052893	0.11567	0.053008	0.053162	0.700704	0.138749	-0.06647	0.150775	0.076242
14	1	0.824021	-0.39275	0.043658	0.224066	0.301007	0.141251	0.199959	0.43938	0.361069	-0.14166
40	1	0.484495	0.166728	0.257264	-0.09262	0.184133	0.375752	0.092505	0.14028	-0.00146	0.39292
5	1	0.779495	0.307595	-0.0298	0.01361	-0.0709	0.487939	-0.06171	0.081372	0.515092	-0.02269
29	1	0.749079	0.13031	0.025452	-0.06491	0.160068	0.685889	-0.08852	0.146789	0.193278	0.062563
39	1	0.920619	-0.03263	0.18964	-0.14811	0.070482	0.685384	-0.01609	-0.05405	0.38551	-0.00075
17	1	0.35589	-0.26513	0.242862	0.095926	0.570448	0.309034	0.403472	0.104287	0.071918	0.111288
7	1	0.763556	0.143893	0.105391	0.089654	-0.1025	0.451357	-0.03776	0.331516	0.179254	0.075635
21	1	0.522084	0.149549	0.0309	0.066185	0.231283	0.399993	-0.21871	-0.11248	0.158447	0.772753
11	1	0.835459	0.238174	0.048518	0.032335	-0.15448	0.497856	-0.04297	-0.04258	0.190662	0.397035
62	2	-0.04157	0.730106	0.426604	0.131537	-0.24668	0.122067	1.02157	0.086868	-0.08017	-0.15033
59	2	0.152838	1.18709	-0.264	0.143662	-0.21959	-0.05759	1.05177	0.109384	0.037655	-0.14122
56	2	-0.03525	0.970161	-0.06383	-0.32491	0.453824	-0.03647	0.686684	0.145056	0.204354	0.000375
72	2	-0.22375	0.783832	0.146678	0.018156	0.275085	0.116799	1.0271	-0.2666	0.286347	-0.16364
44	2	-0.09288	0.727889	0.269895	0.137202	-0.04211	0.147013	0.545495	0.112	-0.04315	0.238644
45	2	0.347346	0.741115	0.135176	0.125903	-0.34954	0.094517	0.533886	0.274407	0.117252	-0.02006
63	2	-0.36978	1.28297	-0.04277	-0.06046	0.190034	-0.16869	0.660997	0.131911	0.044855	0.330929
41	2	0.091552	0.648227	-0.05374	0.168059	0.1459	0.277809	0.25902	0.054423	0.39652	0.012227
51	2	0.644283	0.636612	-0.11202	0.144169	-0.31304	0.590714	0.520661	0.124869	-0.04516	-0.19109
79	2	-0.14528	1.00797	-0.11014	-0.08349	0.33095	0.043626	0.830048	-0.22954	0.174905	0.180957
48	2	0.093244	0.806417	0.158316	0.032828	-0.0908	0.273266	0.426119	-0.03429	0.238599	0.096309
76	2	0.215001	0.64998	-0.00053	0.156229	-0.02068	0.223191	0.803306	0.158541	-0.13873	-0.04631
68	2	0.475353	0.416003	0.127999	0.220442	-0.2398	0.258743	0.386188	0.33079	0.133246	-0.10897
55	2	0.255242	0.728562	-0.0656	0.020237	0.061564	0.191755	0.574394	0.247484	-0.00644	-0.00719
61	2	-0.11909	0.839396	0.371052	-0.11285	0.021491	-0.10986	0.854965	0.073029	0.246203	-0.06433
74	2	-0.03667	0.748646	0.26137	0.138367	-0.11171	0.098108	0.913654	0.176819	-0.24581	0.05723
70	2	-0.17943	0.79752	0.329873	0.066332	-0.01429	-0.20305	0.703854	0.007073	0.3316	0.160528
54	2	0.350763	0.672578	0.058782	0.025589	-0.10771	0.194798	0.698866	0.083076	0.056571	-0.03331
66	2	-0.04242	1.17332	-0.03673	-0.18382	0.089651	-0.19775	1.03858	0.032758	-0.08808	0.214487

64	2	0.229881	0.496512	0.386745	-0.16392	0.050785	-0.19898	0.63483	-0.02959	0.443783	0.149952
57	2	-0.33496	1.00717	0.167775	0.130083	0.02993	-0.10669	0.757446	0.033158	0.130011	0.186079
75	2	-0.08374	0.941284	0.076049	0.170675	-0.10427	0.225478	0.873941	0.263909	-0.22598	-0.13735
47	2	0.066313	0.754041	0.268593	-0.02654	-0.0624	0.115474	0.450232	0.189593	0.113326	0.131374
77	2	-0.04363	0.761669	-0.11931	0.25707	0.144197	-0.15214	0.817218	0.348347	-0.17827	0.16484
53	2	0.194256	0.910701	-0.17604	0.19154	-0.12045	-0.00227	0.668696	0.17643	0.108807	0.048336
50	2	0.04544	0.727227	0.183682	-0.17719	0.220843	0.012458	0.293972	0.162924	0.251016	0.279631
101	3	0.40222	-0.1916	0.665743	0.095743	0.027897	-0.01826	0.092791	0.860533	0.04707	0.01787
91	3	0.050626	0.349324	0.664841	-0.07552	0.010732	0.321047	0.344804	0.342137	-0.25599	0.248005
90	3	-0.11118	0.036083	0.590385	0.046121	0.438588	-0.07006	0.004082	0.016493	0.507968	0.541518
87	3	0.377794	-0.02948	0.561324	0.100185	-0.00982	0.279329	0.30415	0.178417	-0.00193	0.240028
99	3	0.106675	0.265505	0.687359	0.136411	-0.19595	0.003281	0.419573	0.578598	0.222579	-0.22403
114	3	0.153798	-0.00585	0.976027	0.162508	-0.28648	0.367362	-0.12615	0.831369	0.056636	-0.12922
116	3	0.213405	0.311917	0.87153	0.146985	-0.54384	0.210367	-0.01888	0.925147	-0.10521	-0.01142
102	3	-0.24923	0.093465	0.601489	0.265275	0.289002	-0.05079	0.144494	0.587784	0.155995	0.162522
104	3	0.192867	0.049052	0.783877	-0.06439	0.038596	-0.01973	-0.09134	1.14548	-0.15555	0.121145
83	3	0.050315	0.154558	0.756963	-0.01294	0.051106	0.159842	0.414256	0.349952	-0.06163	0.137583
110	3	0.016735	0.010285	0.769619	0.161819	0.041543	0.063131	-0.07938	0.96968	0.15724	-0.11067
108	3	0.186493	0.229437	0.506085	0.111445	-0.03346	0.118982	0.053101	0.967672	-0.09879	-0.04096
81	3	-0.01493	0.098416	0.553611	0.136984	0.225918	-0.15755	0.511106	0.452294	-0.08315	0.277305
107	3	0.193581	-0.06544	0.763858	0.059033	0.048966	0.111284	-0.01864	0.819592	0.19954	-0.11178
112	3	0.104003	0.057247	0.733996	0.111805	-0.00705	0.307486	-0.2479	0.85306	0.25336	-0.16601
113	3	0.148045	0.246981	1.10105	-0.24635	-0.24972	0.411263	-0.01376	0.698304	0.074322	-0.17013
97	3	-0.25161	-0.04437	0.671082	0.363019	0.261882	-0.43797	-0.08966	1.10657	0.638417	-0.21736
85	3	-0.03204	0.101307	0.776261	0.151042	0.003434	-0.07735	0.242919	0.660144	0.046679	0.127606
109	3	0.082844	0.166026	0.429746	-0.05043	0.371811	0.107377	0.006655	0.726918	0.034683	0.124367
105	3	-0.09595	-0.03172	0.882791	0.005487	0.239394	-0.3197	-0.04358	1.10165	-0.10224	0.363873
100	3	0.032304	0.093779	0.530235	0.134147	0.209536	-0.21646	0.443238	0.561869	0.229765	-0.01841
111	3	0.074247	0.087387	0.646762	0.068242	0.123362	-0.00938	-0.09024	0.78358	0.133732	0.182306
96	3	0.191727	0.192212	0.580035	0.007825	0.028201	0.256177	0.541524	0.471902	-0.17462	-0.09498
106	3	0.050868	0.149364	0.604675	0.163614	0.03148	0.159165	0.059674	0.500099	0.019276	0.261786
152	4	0.009381	-0.08712	0.15775	0.737186	0.182802	0.276584	0.197537	0.021736	0.424983	0.07916
121	4	0.005379	0.009133	-0.07445	1.06713	-0.00719	0.493186	0.130033	-0.35927	1.08392	-0.34787
125	4	-0.05214	0.206009	-0.05271	1.04945	-0.15061	-0.05395	0.087723	0.095415	0.579286	0.291526
128	4	-0.07486	-0.17848	-0.16342	1.22494	0.191834	0.285048	0.142342	-0.20754	0.636468	0.143678
145	4	-0.08586	-0.26477	0.639993	0.862846	-0.15221	0.456203	-0.20059	0.332247	0.452308	-0.04016
156	4	-0.03336	0.226259	0.090941	0.526981	0.189179	0.180268	0.288565	-0.13252	0.675792	-0.01211
126	4	-0.14715	0.090178	-0.02761	1.18198	-0.09741	0.121979	0.069777	0.019933	0.438508	0.349803
131	4	0.125178	0.263303	-0.31449	0.882893	0.043117	0.270321	0.208174	-0.00236	0.537076	-0.01321
117	4	-0.33959	-0.14272	0.628849	0.928172	-0.07471	0.263855	0.116175	0.034183	0.919687	-0.3339
122	4	0.03213	-0.10693	0.063161	0.953765	0.057877	0.198071	0.145504	-0.22445	1.05689	-0.17602
149	4	-0.00561	0.166942	-0.00982	0.728777	0.119704	0.432974	-0.05196	0.011385	0.405097	0.202508
153	4	0.166656	-0.09829	0.12777	0.809017	-0.00515	0.32016	0.289623	0.013141	0.496249	-0.11917
146	4	-0.04769	-0.17825	0.114015	0.863997	0.247922	0.297663	0.020057	0.09117	0.394689	0.196421
123	4	0.09845	0.0601	0.114198	0.854487	-0.12724	0.460215	0.032564	0.23864	0.345317	-0.07674
139	4	0.101179	-0.21024	0.261084	0.723996	0.123984	0.328874	0.091764	0.130898	0.563951	-0.11549
140	4	0.117096	0.237697	-0.06926	0.580105	0.134363	0.120149	0.066251	0.211565	0.475007	0.127028
137	4	-0.17778	0.161555	0.083127	0.749435	0.183667	0.113449	0.035694	0.311539	0.417485	0.121833
144	4	0.078488	-0.10868	0.193729	0.846919	-0.01046	0.271379	0.096911	0.279194	0.272949	0.079567

130	4	0.204431	-0.06098	0.022312	0.87758	-0.04334	0.047004	0.245566	0.167945	0.502738	0.036746
118	4	0.213605	0.007883	0.283032	0.740024	-0.24454	0.162014	0.195571	0.179614	0.732248	-0.26945
148	4	-0.04982	0.356595	-0.29999	0.79173	0.201489	0.373479	-0.18129	-0.01322	0.657574	0.163458
134	4	0.000119	0.364539	0.013908	0.141564	0.47987	0.036864	-0.04771	0.098237	0.730187	0.182419
141	4	-0.11283	0.272333	0.1083	0.581231	0.150969	0.11524	0.067685	0.305041	0.468665	0.043368
136	4	0.054555	0.157556	0.3653	0.246958	0.175631	0.167492	0.026491	0.078676	0.741187	-0.01385
154	4	-0.20375	0.101233	0.191161	0.815364	0.095993	0.266086	0.196289	0.03509	0.374905	0.12763
129	4	-0.01074	-0.09995	-0.46884	1.48042	0.099114	0.280675	-0.00548	0.021853	0.685564	0.017389
192	5	0.140281	0.183964	-0.27268	0.229655	0.718775	0.164807	0.175391	0.050719	-0.21937	0.828448
171	5	0.055632	-0.14854	0.132241	0.189486	0.771181	0.073922	-0.09908	0.058012	0.219321	0.747826
169	5	0.050046	0.019033	0.434673	0.017817	0.47843	0.138617	0.104355	0.065215	0.301374	0.390439
162	5	0.264524	-0.08176	0.028901	-0.0339	0.822235	-0.0542	-0.17063	0.134659	0.240985	0.849187
182	5	0.375921	-0.10234	-0.15011	0.110149	0.766379	0.642627	-0.08912	0.043086	-0.05534	0.458747
190	5	0.013491	-0.23269	0.273567	-0.01266	0.958294	0.185355	0.353201	0.122582	-0.02282	0.361683
167	5	0.273735	-0.12844	-0.1404	0.156965	0.838139	0.332131	-0.25853	0.041558	0.173106	0.711734
178	5	0.077011	0.21976	-0.0003	-0.16501	0.868544	-0.13784	0.354722	0.075337	-0.25449	0.962279
196	5	0.254507	0.176587	-0.27525	0.183622	0.660533	0.458181	-0.02819	0.186289	-0.1648	0.548519
165	5	-0.01963	-0.21474	0.438073	0.207403	0.58889	0.212717	-0.19759	0.022585	0.114933	0.847358
187	5	0.246456	-0.06975	-0.19037	0.150165	0.863495	0.217895	0.340789	-0.2662	0.136499	0.571013
191	5	0.426344	-0.11383	-0.22459	0.131614	0.780454	0.443461	0.178945	-0.14975	-0.13416	0.6615
184	5	0.364176	-0.02773	0.025687	-0.15664	0.794504	0.010313	0.507107	-0.1328	-0.16782	0.7832
161	5	0.172221	0.068629	0.027236	0.050804	0.681111	0.162926	-0.22546	0.107469	0.176933	0.778128
180	5	0.190834	-0.06042	0.271259	-0.03826	0.636586	0.21869	0.13309	0.027671	-0.08759	0.708134
175	5	0.177572	0.108944	0.045538	0.00484	0.663105	-0.01055	-0.14483	0.273324	-0.10352	0.98557
179	5	0.308914	-0.02874	0.030893	0.061233	0.627702	-0.06912	0.277867	0.109088	-0.20176	0.883919
168	5	-0.10467	-0.11812	0.39407	0.052475	0.776252	0.050152	0.147282	0.151499	0.148024	0.503043
189	5	0.352452	-0.0796	-0.16137	0.126579	0.761937	0.477356	0.066614	0.071232	-0.00996	0.394762
173	5	-0.07841	0.059287	0.358204	-0.13388	0.794795	0.019687	-0.35315	0.225978	0.2982	0.809284
194	5	-0.15284	0.246629	-0.12077	0.196382	0.830601	-0.0437	0.059501	0.188858	-0.09578	0.891119
195	5	-0.10383	0.213231	-0.05033	0.232343	0.708586	0.022775	0.096065	0.174124	-0.19044	0.897477
170	5	0.171899	0.10713	-0.01997	0.134415	0.606522	0.12695	0.115861	-0.05725	0.321084	0.493357
174	5	0.188584	0.000376	0.10872	0.087206	0.615114	0.058882	-0.06042	0.085714	-0.12471	1.04054
163	5	0.104197	-0.01703	0.076145	0.044394	0.792292	0.196367	-0.08793	-0.22785	0.351772	0.767644
181	5	0.164439	-0.00869	0.07606	-0.06254	0.830735	0.070794	-0.00776	0.129801	-0.00511	0.812272

Table S13

Sample number	Class label	FT-MIR-Bo tree votes (Validation set)					NIR-Bo tree votes (Validation set)				
		Class 1	Class 2	Class 3	Class 4	Class 5	Class 1	Class 2	Class 3	Class 4	Class 5
4	1	0.629762	0.156704	0.15242	0.031494	0.029619	0.307787	0.167566	0.308963	0.293323	-0.07764
6	1	0.415001	0.223477	0.095563	0.097393	0.168566	0.200131	0.070401	0.218816	0.390178	0.120475
8	1	0.475554	0.067417	0.123989	0.117443	0.215598	0.508176	0.028748	0.125476	0.13069	0.206911
10	1	0.767028	0.170241	-0.04157	0.080682	0.023618	0.368855	-0.12322	0.257061	-0.00348	0.500792
13	1	0.511023	-0.00226	0.286418	0.238933	-0.03411	0.121127	0.234893	0.337104	0.358039	-0.05116
15	1	0.314626	0.261308	0.158624	0.165032	0.100409	-0.11544	0.343309	0.517012	0.046146	0.208975
22	1	0.548241	-0.01194	0.022058	-0.01219	0.453829	0.389583	0.062907	0.028703	0.379909	0.138898
24	1	0.392129	-0.03528	0.025036	0.159837	0.458277	0.373572	0.106987	-0.15076	0.3475	0.322704
28	1	0.56932	0.043479	0.259877	-0.03792	0.165243	0.609429	-0.08151	0.189445	0.297188	-0.01455
30	1	0.740108	-0.02892	0.092548	-0.04427	0.240541	0.384674	-0.00159	0.060243	0.179015	0.377661
32	1	0.422644	-0.15828	0.365592	-0.16373	0.533774	0.492653	-0.04542	-0.10022	0.396776	0.256205
34	1	1.18641	0.00861	-0.20475	0.101374	-0.09165	0.594252	-0.00536	0.118082	0.144429	0.148597
35	1	0.643108	-0.14217	0.205397	-0.11347	0.407136	0.627805	-0.14659	-0.2026	0.472333	0.249044
37	1	0.435375	0.012341	0.445422	-0.15844	0.265298	0.613513	-0.10498	0.095089	0.12877	0.26761
42	2	-0.17698	0.564548	0.170932	0.401656	0.039841	0.216785	0.206888	0.399226	0.172261	0.00484
43	2	0.138327	0.632912	0.020704	0.174371	0.033686	0.156819	0.076719	0.365625	0.207951	0.192886
46	2	0.029609	0.819205	-0.01264	-0.01134	0.175173	-0.02084	0.228136	0.119707	0.338235	0.334763
49	2	0.173349	0.792731	0.096172	-0.0458	-0.01646	0.095313	0.47253	0.1838	-0.05911	0.307468
52	2	0.110338	0.932086	-0.08312	-0.05907	0.099759	-0.01079	0.431762	0.447679	-0.01648	0.147826
58	2	0.001005	0.708155	-0.0952	0.37901	0.007027	-0.1028	0.481512	0.351669	0.225162	0.044455
60	2	-0.09653	0.911193	-0.0083	0.217257	-0.02362	-0.00202	0.860069	0.247373	-0.07607	-0.02935
65	2	-0.11434	0.98335	0.318661	-0.19348	0.005815	0.005442	0.804785	-0.16551	0.441017	-0.08573
67	2	0.14692	0.538651	0.288411	0.057294	-0.03128	-0.01135	0.754658	0.027255	-0.04353	0.272965
69	2	-0.11804	0.496712	0.409843	0.187517	0.02397	-0.03578	0.75466	0.262139	-0.00684	0.025816
71	2	-0.16994	1.1525	-0.06394	-0.10786	0.189234	0.10073	1.07438	-0.12256	0.063571	-0.11612
73	2	-0.15802	0.574479	0.194251	0.27897	0.110322	0.088054	0.62897	0.130155	0.31394	-0.16112
78	2	-0.10685	0.738584	0.253039	-0.01215	0.127374	-0.03223	0.73926	0.267755	-0.00054	0.025757
80	2	-0.04079	0.621186	0.194977	0.118454	0.10617	-0.1138	0.630779	0.178933	-0.05386	0.357954
82	3	0.164088	-5.3E-05	0.589285	0.016657	0.230023	-0.1063	0.392379	0.532632	0.012779	0.168512
84	3	0.086168	0.085206	0.664889	-0.00841	0.172143	0.099745	0.408157	0.147189	0.006433	0.338476
86	3	0.106927	0.02547	0.58515	0.177995	0.104458	0.149647	0.299194	0.145334	0.054271	0.351553
88	3	-0.13607	0.110981	0.354281	0.190602	0.480204	-0.14472	0.269603	0.302421	0.232157	0.340535
89	3	-0.15083	0.165057	0.547706	0.073496	0.364575	0.106826	0.05274	0.080654	0.419883	0.339897
92	3	0.102585	0.204706	0.40317	0.254057	0.035482	0.35624	0.33948	-0.05496	0.003999	0.355245
93	3	0.11898	0.228175	0.546778	0.166298	-0.06023	0.472254	0.108381	0.307666	-0.04646	0.158159
94	3	0.072851	0.06303	0.5283	0.083358	0.252461	0.214212	-0.09705	0.431268	-0.01115	0.462721
95	3	0.183533	0.175816	0.412299	0.141226	0.087127	0.49337	0.150793	0.142016	-0.09715	0.310971
98	3	-0.22109	0.528953	0.487374	0.326287	-0.12153	-0.21984	0.349075	0.563898	0.280022	0.026843
103	3	-0.16816	-0.32285	1.01796	0.305805	0.167241	0.10143	-0.18935	0.744231	0.551595	-0.20791
115	3	-0.03234	0.211332	0.788816	0.152327	-0.12014	0.211029	0.058437	0.592262	0.014842	0.12343
119	4	-0.16514	0.255755	0.51689	0.827705	-0.43521	0.227668	0.166744	-0.00534	0.733962	-0.12303
120	4	0.029998	0.152035	0.226092	0.714595	-0.12272	0.222679	-0.04352	-0.01973	0.938661	-0.09809
124	4	0.343787	0.103356	-0.10593	0.751433	-0.09264	0.364991	-0.17653	0.037715	0.766154	0.007668
127	4	0.192168	0.25253	0.058942	0.764868	-0.26851	0.170147	0.111676	0.016722	0.553105	0.148351
132	4	0.033669	-0.11329	-0.15745	1.20771	0.029355	0.233383	0.185117	0.037576	0.645181	-0.10126
133	4	-0.01676	0.018646	0.046217	1.1792	-0.2273	0.137093	0.105997	0.217569	0.641476	-0.10214

135	4	0.151166	0.053012	0.052215	0.215015	0.528592	0.233335	-0.00722	0.099297	0.588103	0.08648
138	4	-0.31897	-0.00077	0.134446	0.759467	0.425826	0.08071	0.124156	0.073174	0.587417	0.134542
142	4	0.216379	-0.07555	0.036622	0.580442	0.242105	0.127731	0.195347	0.218973	0.38314	0.074809
143	4	-0.01893	0.088441	-0.00291	0.755423	0.177978	0.184001	0.131215	-0.03076	0.530959	0.184582
147	4	-0.07425	-0.10258	0.012752	0.859819	0.304257	0.393986	-0.05714	0.106137	0.535503	0.021513
150	4	0.142024	-0.16211	0.055356	0.825594	0.139134	0.330206	0.023726	-0.04895	0.557194	0.137821
151	4	0.015852	-0.06506	0.233389	0.666068	0.14975	0.402202	-0.08472	-0.15776	0.454012	0.386263
155	4	-0.18109	0.095237	0.187878	0.739694	0.158278	0.320344	0.150789	0.014952	0.521896	-0.00798
157	5	0.048106	0.066874	0.189857	0.067235	0.627928	-0.07262	-0.12361	0.298315	0.377259	0.520654
158	5	0.103057	0.100775	0.054129	-0.04254	0.784574	-0.02953	0.019412	-0.06286	0.356314	0.716665
159	5	0.08978	-0.10048	0.086687	0.091168	0.83284	0.090911	0.021634	0.041634	0.361476	0.484345
160	5	0.031938	0.095837	-0.06274	0.120549	0.814412	0.095276	-0.01756	-0.048	0.340985	0.629299
164	5	0.137295	0.16223	-0.045	0.056049	0.689422	0.373777	-0.26413	-0.14471	0.303495	0.73157
166	5	0.072367	-0.1338	0.10431	0.149136	0.80799	0.201636	-0.18188	-0.15515	0.470376	0.665023
172	5	0.093377	0.239185	0.142589	0.021955	0.502894	0.098369	-0.02935	-0.07322	0.273267	0.730936
176	5	0.036184	0.047022	0.314247	0.140994	0.461552	0.222228	-0.21918	-0.02295	0.059157	0.960747
177	5	0.445504	0.068137	0.040711	-0.27354	0.719192	-0.02927	0.204077	0.397336	-0.41078	0.838636
183	5	0.382457	-0.36982	0.14847	-0.12694	0.965834	0.140804	0.366689	0.027638	-0.09656	0.561435
185	5	0.152328	-0.06346	-0.00948	0.100479	0.820131	0.039153	0.238374	0.058746	-0.05372	0.71745
186	5	0.291032	-0.07189	0.038232	-0.05992	0.802553	0.220176	0.160278	0.074505	0.00877	0.536271
188	5	0.081942	-0.19676	-0.04319	0.300826	0.857179	0.001247	0.195605	-0.00257	-0.01362	0.819339
193	5	-0.03471	0.151421	-0.33448	0.199074	1.0187	-0.071	0.181324	0.022071	-0.06936	0.936964

Table S14

	Tree vote fractions of RF model					Ensemble decision
	Class 1	Class 2	Class 3	Class 4	Class 5	
Class 1, sample 6						
FT-MIR-PCs	0.247	0.150	0.084	**0.321**	0.198	
NIR-PCs	**0.360**	0.183	0.132	0.281	0.044	
Maximum	**0.360**	0.183	0.132	0.321	0.198	Class 1
Minimum	0.247	0.150	0.084	**0.281**	0.044	Class 4
Product	**0.303**	0.167	0.108	**0.301**	0.121	Class 1, 4
Average	**0.089**	0.027	0.011	**0.090**	0.009	Class 1, 4
Majority vote						**Class 1, 4**
Class 1, sample 4						
FT-MIR-PCs	**0.501**	0.105	0.069	0.264	0.062	
NIR-PCs	0.333	0.073	0.141	0.109	**0.345**	
Maximum	**0.501**	0.105	0.141	0.264	0.345	Class 1
Minimum	**0.333**	0.073	0.069	0.109	0.062	Class 1
Product	**0.417**	0.089	0.105	0.186	0.203	Class 1
Average	**0.167**	0.008	0.010	0.029	0.021	Class 1
Majority vote						**Class 1**
Class 1, sample 31						
FT-MIR-PCs	0.136	0.105	0.209	0.162	**0.387**	
NIR-PCs	0.227	0.190	**0.353**	0.123	0.107	
Maximum	0.227	0.190	0.353	0.162	**0.387**	Class 5
Minimum	0.136	0.105	**0.209**	0.123	0.107	Class 3
Product	0.182	0.147	**0.281**	0.143	0.247	Class 3
Average	0.031	0.020	**0.074**	0.020	0.041	Class 3
Majority vote						**Class 3**
Class 3, sample 114						
FT-MIR-PCs	0.217	**0.354**	0.122	0.185	0.122	
NIR-PCs	0.085	0.180	**0.407**	0.258	0.070	
Maximum	0.217	0.354	**0.407**	0.258	0.122	Class 3
Minimum	0.085	**0.180**	0.122	**0.185**	0.070	Class 2, 4
Product	0.151	**0.267**	**0.264**	0.221	0.096	Class 2, 3
Average	0.018	**0.064**	0.050	0.048	0.008	Class 2
Majority vote						**Class 2**

Table S15

Source	Region	Location	Sample size
Wild	Central Yunnan	Songming, Kunming	20
		Jiangchuan, Yuxi	20
	Western Yunnan	Weixi,Diqing	20
		Fugong, Nujiang	20
	Northwest Yunnan	Longyang, Baoshan	16
		Yunlong, Dali	20
	Southeast Yunnan	Mingzi, Honghe	20
		Jianshan, Wenshan	20
	Southwest Yunnan	Mojiang, Pu’er	20
		Yunxian, Lincang	20
